# Discrete Logic Modelling Optimization to Contextualize Prior Knowledge Networks Using PRUNET

**DOI:** 10.1371/journal.pone.0127216

**Published:** 2015-06-09

**Authors:** Ana Rodriguez, Isaac Crespo, Anna Fournier, Antonio del Sol

**Affiliations:** Luxembourg Centre for Systems Biomedicine (LCSB), University of Luxembourg, Luxembourg; University of Erlangen-Nuremberg, GERMANY

## Abstract

High-throughput technologies have led to the generation of an increasing amount of data in different areas of biology. Datasets capturing the cell’s response to its intra- and extra-cellular microenvironment allows such data to be incorporated as signed and directed graphs or influence networks. These prior knowledge networks (PKNs) represent our current knowledge of the causality of cellular signal transduction. New signalling data is often examined and interpreted in conjunction with PKNs. However, different biological contexts, such as cell type or disease states, may have distinct variants of signalling pathways, resulting in the misinterpretation of new data. The identification of inconsistencies between measured data and signalling topologies, as well as the training of PKNs using context specific datasets (PKN contextualization), are necessary conditions to construct reliable, predictive models, which are current challenges in the systems biology of cell signalling. Here we present PRUNET, a user-friendly software tool designed to address the contextualization of a PKNs to specific experimental conditions. As the input, the algorithm takes a PKN and the expression profile of two given stable steady states or cellular phenotypes. The PKN is iteratively pruned using an evolutionary algorithm to perform an optimization process. This optimization rests in a match between predicted attractors in a discrete logic model (Boolean) and a Booleanized representation of the phenotypes, within a population of alternative subnetworks that evolves iteratively. We validated the algorithm applying PRUNET to four biological examples and using the resulting contextualized networks to predict missing expression values and to simulate well-characterized perturbations. PRUNET constitutes a tool for the automatic curation of a PKN to make it suitable for describing biological processes under particular experimental conditions. The general applicability of the implemented algorithm makes PRUNET suitable for a variety of biological processes, for instance cellular reprogramming or transitions between healthy and disease states.

## Introduction

The wealth of experimental data from high-throughput technologies in different areas of biology allows such data to be incorporated as networks of interactions. Gene regulatory networks (GRNs) can be reconstructed based on knowledge resources such as literature or specific databases, the so called prior knowledge (PKN,) or purely from specific experimental datasets by inferring functional interactions between statistically correlated levels of biological entities such as genes or proteins.

PKNs are inclusive by nature because they usually merge altogether interactions previously described in different biological contexts such as different cell types, tissues or experimental conditions in the attempt to collect all relevant biological events. In addition, they suffer from undetermined network incompleteness, which is biased due to historical reasons. PKNs are available in specific databases [1–3] or can be constructed using dedicated software tools. A number of both commercial and free software resources are available to assist with the reconstruction of PKNs, for example Pathway Studio (www.elsevier.com/online-tools/pathway-studio), Ingenuity Pathway Analysis (www.ingenuity.com), Metacore (www.thomsonreuters.com/metacore), Transfac (www.biobase-international.com/product/transcription-factor-binding-sites) and GenMania (www.genemania.org).

On the other hand, there are a number of methods to infer networks purely from data through reverse engineering based on statistical correlation or mutual information, such as the structural equation model (SEM) [4], the graphical Gaussian model (GGM) [5–7], the algorithm for the reconstruction of accurate cellular networks (ARACNE) [8,9] and context likelihood of relatedness (CLR) [10]. In general, these methods are comprehensive (to the same extent as the experimental technique used to collect the data) and strongly contextualized to the experimental conditions. However, these networks lack directionality and usually yield a number of ‘false’ interactions because of the statistical co-occurrence of events that are not due to cause-effect relationships and indirect correlations (if ‘A’ has an effect on ‘B’ and ‘B’ has an effect on ‘C’, we may observe a statistical correlation between ‘A’ and ‘C’ which is not a direct cause-effect relationship). Despite the attempt to remove these indirect interactions exploiting partial correlations [5–7,11], conditional mutual information [12,13] or data processing inequality [8,9], this important issue still hinders the elucidation of the underlying mechanisms (the causal network) and decreases the reliability of the predictions of models based on these networks. Some network inference methods tackle the problem of directionality. For example, Nested Effects Models (NEMS) [14–18] use the nested structure of phenotypic effects caused by different knockdowns to infer a hierarchy of genes under the assumption that the knockdown of a gene B produces a subset of the effects observed after the knockdown of gene A if gene B is downstream in the hierarchy. Consequently, an inferred interaction between these two genes has the direction from A to B. However, NEMs have severe limitations in regard to large networks and cannot handle combinatorial knockdowns and complex network topologies (with feedback and feed-forward loops). To address these limitations Deterministic Effects Propagation Networks (DEPNs) [19] were developed. DEPNs assume deterministic signaling in the underlying network and alternative network topologies are scored based on the measurement distribution of active and inactive proteins, which are estimated from data using maximum likelihood inference or maximization of the posterior distribution. A limitation of DEPN models is that multiple incoming edges to a node are always integrated in ‘AND’ functions; the model does not consider other logic relationships.

In general, neither of these two strategies (PKNs and network inference from data) yields networks that can be used directly to construct reliable dynamical models to describe or simulate biological processes, especially when the purpose of the model is to predict input-output relationship such as responses to single or multiple perturbations (for example, cellular responses to specific growth factors, cytokines or transcription factors).

In an attempt to overcome the drawbacks mentioned above, methods that combine information from the literature and experimental data to reconstruct biological networks have been proposed [20–31]. Combinatorial optimization methods have been proposed to address the network inference problem from perturbation data and PKNs. Using an integer programming strategy, Ourfali et al. [26] proposed a method that combines expression measurements after knockout experiments with a PKN to reconstruct regulatory pathways in yeast. Similarly, Lan et al. [27] linked genetic and transcriptomics data with previously known interactions. However, Hashemikhabir et al. [28] recently showed that even with an almost correct PKN, finding the minimum number of network topology changes that makes the model consistent with experimental data is an NP complete problem. The complete enumeration of the solution space becomes infeasible for n>6 [14,32]. Knapp et al [29] proposed a formulation of the network inference problem as a non-integral linear program that can be solved with polynomial time solvers. Edges have continuous weights, and the decision about the inclusion of a given edge in the final network is based on a heuristic decision using a threshold discretization. Prior knowledge about known interactions or the nature of nodes (for example, if they are ligands or receptors) can be used to formulate additional constraints. However, these models always assume ‘OR’ functions between multiple regulators of the same node, and the method cannot handle ‘AND’ logic functions because the model becomes nonlinear.

Within the group of linear programming-based approaches, it is worth mentioning SigNetTrainer [30], a freely available toolbox that uses integer linear programming on PKNs to encode constraints on the qualitative behaviour of the nodes. SigNetTrainer looks for the optimal subgraph of the given PKN that best reflects the measurements from a set of experimental scenarios. Within the Results and discussion section, SigNetTrainer is compared with the method proposed in this work.

Some heuristic approaches based on discrete logic modelling have been proposed to address the computational limitations of combinatorial optimization methods. These heuristic methods can be roughly classified into those designed to model the dynamics of signalling pathways [20, 21], and those oriented to the description of the stable steady-states of biological networks (attractors), for instance stable expression profiles of GRNs. The first group of methods, which includes freely available implementations such as CellNOptR [25] and MetaReg [24], requires multiple perturbation (stimulus-response) experiments to optimize and/or expand canonical signalling pathways. CellNOptR takes as the input a PKN that is translated to a superstructure of Boolean networks within which the optimal submodel fitting perturbation data at best is identified. CellNOptR was compared with the method proposed in this work (see Results and discussion section). The MetaReg platform offers a model refinement tool to enhance the fitness between experimental data and model predictions, but it is oriented to optimize just the logic functions and assumes a fixed network topology. Similarly, IRIS [31] uses an iterative approach to map gene expression profile values (both steady-state and time course) into discrete states and a simple probabilistic method to infer the regulatory logic of a given PKN rather than the topology itself.

Methods in the second group (steady-states oriented) [22,23] usually require a smaller number of experimental measurements of the attractor states, such as gene expression data or protein levels, although their predictions are strongly contextualized to these states.

The idea of matching Boolean steady states with observed experimental data as a fitness criterion was initially proposed by Kim et al. [33], who applied this concept to construct, from experimental data, a small model of 10 genes to describe the invasive phenotype acquisition in melanoma cells. Pal et al. [34] proposed a method to generate Boolean networks with a prescribed set of attractors without previous information about connectivity. This prescribed set of attractors is derived from experimental observations assumed as steady states.

Within the group of methods combining PKN and experimental data oriented to the description of steady states, it is worth noting the strategy proposed by Layek et al. [22], which exploits the agreement between observed and computed attractors to assess possible subnetworks in the original PKN, however this strategy lacks an optimization method to improve these subnetworks, whereas Crespo et al. [23] proposed an evolutionary algorithm for this purpose that has been successfully applied in several published works [35–37].

It is also worth mentioning here another related strategy that can be applied to combine PKNs and experimental data, the so-called probabilistic Boolean networks (PBNs) [38]. Basically, PBN is a hybrid approach to describe the dynamics of biological networks that is placed intermediately between purely probabilistic methods, such as Bayesian networks [39], and Boolean networks. PBNs consider alternative Boolean functions for each node, which depend both on the topology and the relationships between co-regulators. The probability that a given subset of Boolean functions is applied when simulating the dynamics of the system is derived from experimental data in a variety of biological contexts. The main difference that distinguishes the method presented in this work and PBNs is that the latter one attempts to integrate different biological contexts in a single model whereas the algorithm presented in this work yields a population of alternative models that are context-specific. These context-specific models can be considered as a special case of the PBNs and require less experimental data and computation. These models cannot address the biological uncertainty but they can be considered as descriptors of the average behavior of the system in a particular biological context.

Here we present PRUNET, a user-friendly software tool to address the contextualization of PKNs using a Booleanized representation of observed expression profiles corresponding to stable phenotypes. Consequently, PRUNET can be included among the heuristic methods based on discrete logic modelling and oriented to the description of the steady states (attractors) of a biological network.

PRUNET differs from its most closely related method, the one proposed by Crespo et al. [23], in that it uses a more efficient optimization strategy; instead of exploring the entire search space of attractors, PRUNET assigns the expression profiles as initial conditions and updates the system until it falls into an attractor, which is compared with the initial conditions. This strategy rests on the assumption that a network state belonging to the basin of attraction of a given attractor is more similar to this attractor (in Hamming distance terms) than to any other attractor in the attractor space. The validation of the utility of this central assumption is the main contribution of this work and it represents a significant improvement upon other previously published algorithms. Following this strategy PRUNET avoids the construction of the reachability graph. In addition, PRUNET encodes the regulatory logic functions as a system of algebraic equations, instead of as a binary decision diagram (whose construction is very computationally demanding for big networks). Using this strategy, PRUNET reduces dramatically the computational time and contextualizes networks of thousands of nodes, whereas the implementation of Crespo et al. [23] and other similar software, such as CellNOptR [25], are limited to a few hundred nodes or less, depending on the complexity of the connectivity.

PRUNET has been entirely implemented using Perl programming language in order to facilitate its modification and its cross-platform use. In addition, PRUNET provides a graphical user interface (GUI) to facilitate the interaction with the user.

Apart from the PKN, the other input that PRUNET requires is a list of up- and down-regulated genes resulting from the comparison between two stable phenotypes. PRUNET is going to transform this list in two vectors or Booleanized phenotypes. For example, if a gene is up-regulated when comparing two given initial and final phenotypes, then it is assumed to be ‘0’ and ‘1’ in the initial and final phenotypes respectively. In other words, a single experiment comparing two stable phenotypes is enough to contextualize the PKN using PRUNET.

Once interactions inconsistent with the observed phenotypes have been removed the resulting contextualized network can be used to predict missing expression values or to simulate transitions between attractors in response to perturbations.

In order to validate the algorithm and to show its general applicability and scalability, we applied PRUNET to four biological examples, namely the epithelial to mesenchymal transition (EMT), T-Helper lymphocytes transdifferentiation (Th1-Th2), the induction of pluripotent stem cells (iPSC) and the differentiation of human embryonic stem cells (hESC) into cardiomyocytes (hESC-cardiomyocyte).

The resulting contextualized networks were used to predict expression values given a set of validation and training genes. We challenged the algorithm through cross-validation using different randomly generated training and validation sets. The results showed that even training sets with only 50% of the genes yield contextualized networks that performed better than randomly generated subnetworks in predicting missing expression values.

Finally, we simulated well-characterized perturbations to evaluate the dynamical behaviour of the best-contextualized networks for each example. The results showed that, although the models were only trained to explain the existence of specific stable states (corresponding to specific cellular phenotypes), a fraction of the contextualized networks also exhibited the expected response under perturbation (20%, 80%, 32% and 36% for EMT, Th1-Th2, iPSC and hESC-cardiomyocyte respectively).

PRUNET constitutes a user-friendly tool conceived not only for bioinformaticians and computational biologists but also for experimentalists. Its general applicability and the balance between the amount of data required to train the models and the accuracy of the predictions makes it suitable in a variety of biological systems and research projects. It can be used to help in both experimental design and hypothesis generation in biological and medical research.

## Methods

### Problem formulation

Given a PKN and a training set of experimental data, find the subnetwork/s of the PKN that best describe the experimental data for an adopted dynamical model.

This model consists of a Boolean network where the state of node *i* is given by the Boolean variable *x*_*i*_ ∊ {0,1}, which corresponds to the gene product activity/concentration. The state of the network is given by the state of all nodes *X* = {*x*_1,…,_*x*_*n*_}, which is updated using a synchronous updating scheme [40][40].

The training set consists on a Booleanized representation of the gene product activity/concentration, where those genes considered active/inactive or up-/down-regulated have a value of ‘1’/’0’. For instance, the differential expression analysis between two conditions (corresponding to stable phenotypes) results in a list of up- and down-regulated genes, and two Booleanized phenotypes can be derived from this list with the activity of these differentially expressed genes for each condition, i.e., up-regulated genes are in ‘0’ at the initial condition and in ‘1’ at the final condition, and the opposite for down-regulated genes. The goodness of the model description of the experimental data is evaluated as follows:

1. Booleanized phenotypes derived from experimental data are introduced in the model as initial states.
2. The model is updated until it reaches an attractor.
3. Reached attractors are compared with the initial states.

A perfect model should have a perfect match between the reached attractors and initial states (Booleanized phenotypes).

The fitness function *f* derived from this proceed uses the normalized Hamming distance *h* to compare *N*_g_ Boolean gene expression values (*x*) between the known phenotypes (*φ*) and the reached steady state (α) of a given subnetwork, and it can be represented as

$$f=\left(1-\frac{\left(\sum_{i=1}^{N_{p}}h_{\varphi_{i}\alpha_{i}}\right)}{N_{p}}\right)$$

with $h_{\varphi \alpha }=\frac{1}{N_{g}}\sum_{j=1}^{N_{g}}\left|x_{j}^{\varphi }-x_{j}^{\alpha }\right|$, where *N*_*p*_ represents the number of Booleanized phenotypes

Given that the fitness function *f* is normalized by the number of Booleanized phenotypes *N*_*p*_, the best and worst possible values are 1 and 0 respectively. The value of *f* represents the fraction of nodes that are correctly explained by the model for all considered Booleanized phenotypes.

### Model formalism

The Boolean model is defined by a set of polynomial functions encoding the regulatory logic as follows:

- And (conjunction): *x* ^ *y* = *x* × *y*
- Or (disjunction): *x* ˅ *y* = *x* + *y-* (*x* × *y*)
- Not (negation): ¬*x* = 1-*x*

For example, the following Boolean network

$$\begin{array}{l} a=b\wedge c\\ b=\neg b\\ c=a\vee b \end{array}$$

is represented as the following system of polynomial functions

$$\begin{array}{l} a\prime =b\times c\\ b\prime =1-b\\ c\prime =a+b-(a\times b) \end{array}$$

Within the context of the method implemented in PRUNET, solving these expressions for a given set of initial states *a*, *b* and *c* (Booleanized phenotype) yields the values in the next (*a'*, *b'* and *c'*). In the examples included in this work we applied a synchronous updating scheme, which assumes that all nodes are updated at the same time. Eventually, the current and next steps coincide because the system has reached a steady state or the system revisits previously visited states in a cyclic fashion. In the first case the reached state is compared with the Booleanized phenotype used as the initial conditions whereas in the second case, when the systems reaches a cyclic attractor the Hamming distance is not calculated and the score of the subnetwork is 0. This strategy differs from the one followed by XPRED [23], where the complete attractor space for each subnetwork was explored in order to find the closest attractor to the Booleanized phenotype, with the consequent increase in computation time and limited network size.

#### Boolean network construction from PKN

The Boolean model is derived from the PKN by adopting a synchronous updating scheme, which assumes that all nodes are updated at the same time (not in a defined or random sequence). The regulatory logic functions adopted to update the network state follow the next rule: a node is considered active if at least one of its activators and none of its inhibitors are active. The node is considered inactive otherwise. The system of regulatory logic functions of the PKN for each example is included in the supplements (S1 Table).

### Model perturbations

Network perturbations were simulated as an instant network state change, where the states of the perturbed genes are changed either from ‘0’ to ‘1’ or from ‘1’ to ‘0’ depending of the initial state. For example, SNAI1, GATA3 and the combination KLF4/POU5F1/SOX2 for EMT, Th1-Th2 and iPSC respectively were swapped from their original inactive to a final active state, i.e., from ‘0’ to ‘1’. In the hESC-cardiomyocyte example, SOX2 and MEF2C were swapped from ‘1’ to ‘0’ and from ‘0’ to ‘1’ respectively. For all cases the network state was freely updated afterwards using a synchronous updating scheme.

### Algorithm

**Fig 1:**
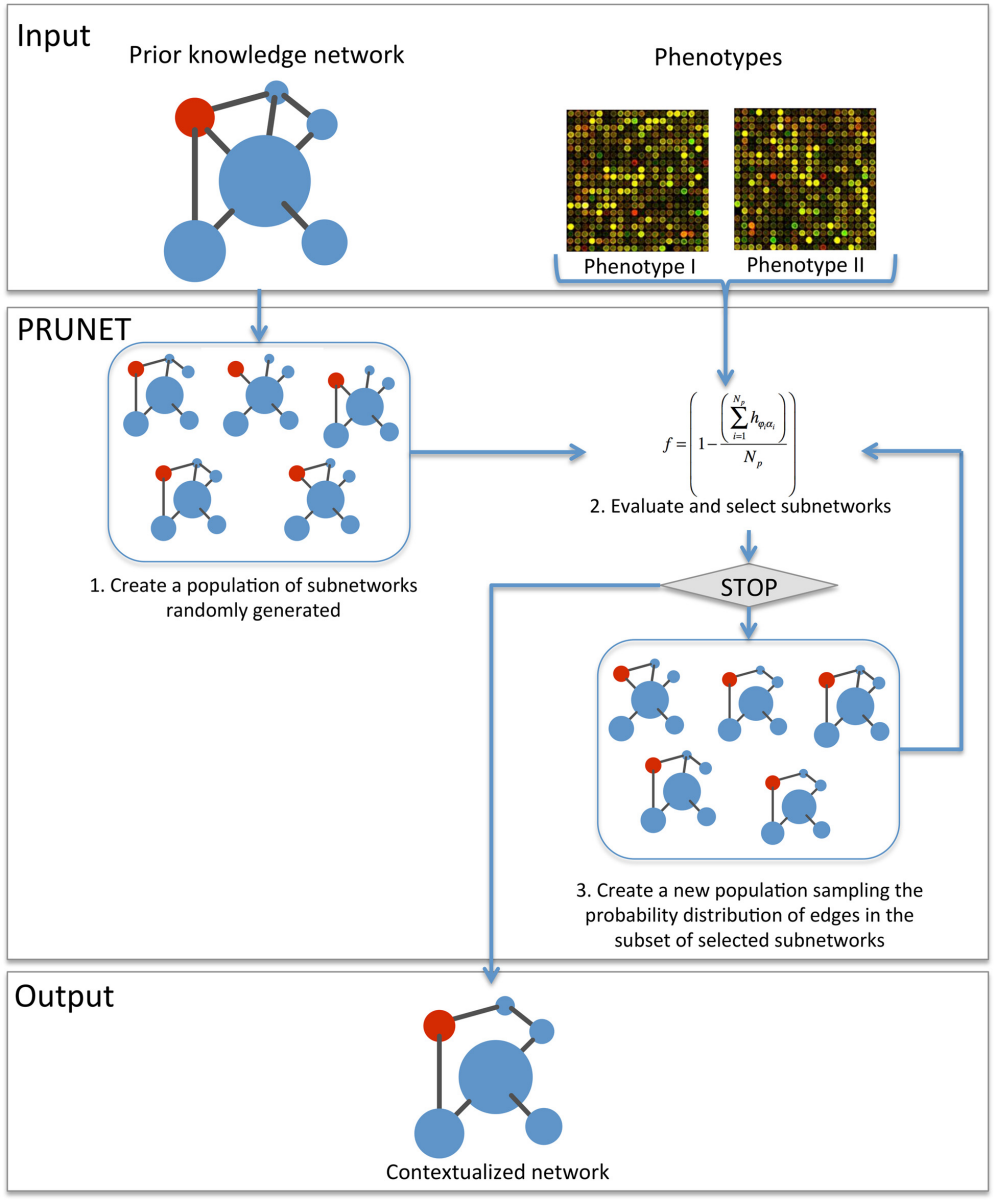
Flow chart of the algorithm. PRUNET takes as the input a prior knowledge gene regulatory network and a Booleanized representation of the gene expression profiles of stable phenotypes. After an iterative network pruning, PRUNET delivers as the output a single (or several) contextualized network(s) optimized to describe the phenotypes according to an adopted dynamical model (Boolean). The fitness function is based on the matching between the predicted (α) and the known gene states (φ) for specific phenotypes.

Finding a good model just by randomly generating subnetworks from the PKN is very unlikely. PRUNET includes an optimization algorithm to search for the best subnetwork. It consists of an estimation of distributions algorithm (EDA), a type of evolutionary algorithm that has been used for different bioinformatics problems [41]. The algorithm applies the fitness function described above (see Problem formulation in Methods section) to train the model in a process that can be described as follows (see Fig 1):

1. Create a population of randomly generated subnetworks. All edges and nodes in these networks must exist in the PKN.
2. Evaluate the fitness function of each subnetwork and select those with the highest score.
3. Create a new population of subnetworks based on those selected in the previous step. To this end, PRUNET samples the probability distribution of each edge within the selected group of subnetworks and decides whether to include this interaction or not. For instance, let us suppose that a specific edge appears with a frequency of 0.80. PRUNET generates a random number between 0 and 1 and uses this frequency as the threshold for making a decision. If the random number is lower or equal to 0.80, the edge is kept. Conversely, if the random number is higher than 0.80, the edge is removed.
4. Repeat step 2. The stop criterion is the maximum number of iterations previously defined by the user, but PRUNET allows the optimization to be stopped at any time and the current results to be collected.

### Complexity of the algorithm

When applying PRUNET to a given system, the computation time depends on the number of iterations and the population size of the evolutionary algorithm (which is selected by the user). However, if we focus on the asymptotic behavior of the algorithm, dropping all optimization parameters and keeping only the largest growing term when increasing the network size, the bottleneck is the identification of the reached attractor from a given initial condition. Given a graph **G**(**V, E**), with **V** being the number of vertices and **E** the number of edges, identifying all of the attractors in **G** can be performed in linear time **O**(**|V|** + **|E|**) by depth-first search algorithm. However, for Boolean functions, there can be an exponential number of vertices (i.e. **|V|** = 2^**N**^) in the graph for **N** Boolean variables, resulting in an exponential space complexity of the problem of identifying all of the attractors in the Boolean state space.

Despite PRUNET not searching for all of the attractors of the system, the worst-case analysis considers a scenario in which all of the states of the Boolean state space have to be evaluated. In other words, the trajectory from the initial conditions (phenotypes) to the final state (reached attractor) passes through all states of the Boolean state space, updating every node at each state, i.e., **O**(**N**x2^**N**^). This hypothetical situation is extreme and unlikely to occur in a real case. Moreover, the optimization itself is going to prevent this situation, which is more likely to occur at the first iterations of the algorithm. In order to avoid such a possibility, PRUNET is implemented in such a way that subnetworks resulting in trajectories longer than a given number of steps (1,000 within this work) are discarded, so that the algorithm to calculate the reached attractor becomes **O**(**N**x1000).

### Examples: input networks and training sets

We have demonstrated the broad applicability and scalability of PRUNET using four examples corresponding to four different biological processes, namely the epithelial to mesenchymal transition (EMT), T-Helper lymphocytes transdifferentiation (Th1-Th2), the induction of pluripotent stem cells (iPSC), and the differentiation of human embryonic stem cells (hESC) into cardiomyocytes (hESC-cardiomyocyte).

The selected examples are not just ordinary PKNs, but are previously published network-based dynamical models manually constructed that correctly describe known phenotypes at the transcriptional level [42–45]. In these networks, nodes represent gene or gene products (such as proteins or microRNAs), and edges represent regulatory effects (up- or down-regulation for positive and negative interactions respectively). These regulatory effects can consist either of direct molecular interaction, such as the effect of a transcription factor over its target, or indirect interactions, such as the observed change in the expression levels due to known or unknown signaling pathways. For validation purposes, these networks that nicely explain the phenotypes used as training set in this work constitute the gold standard assumed correct. These networks were expanded with the addition of interactions between their constitutive genes contained in the ResNet mammalian database from Ariadne Genomics (http://www.ariadnegenomics.com). Such interactions have been extracted from the biomedical literature using Ariadne MedScan technology [46,47], a text mining-based resource that processes sentences from PubMed abstracts. The interactions were collected blindfolded, without the biological context being filtered. Consequently, the interactions have been described in different cell types, biological processes and experimental conditions. It is worth noting here that this is a common practice during the reconstruction of GRNs in the attempt to capture all relevant events. The rationale behind this procedure is to collect as many true interactions as possible despite the fact that some context-dependent interactions may not be active in the specific biological context under study. Consequently, some of the added interactions are consistent with the known phenotypes used to train the model, whereas other added interactions are not.

For our purpose, we blindly added both consistent and inconsistent interactions to evaluate the capacity of PRUNET to remove the latter instances. It is important here to note that all interactions added to the original GRNs are directed and signed (either activating or inhibiting effects), including interactions classified in the database in the categories of “Promoter Binding”, “Expression” and “Direct Regulation”. Interactions in the “Promoter Binding” category indicate that the regulatory gene binds the promoter of the target genes and shows potential regulation in the experiment. “Expression” represents the positive or negative regulatory effect of the regulator on the expression of the target. Finally, “Direct Regulation” indicates that the regulatory gene/protein alters the activity of the target gene/protein by means of physical binding, phosphorylation, methylation or other processes.

Follwing the network expansion described above, the four original networks of EMT, Th1-Th2, iPSC and hESC-cardiomyocyte with 16, 60, 123 and 126 interactions were expanded up to 19, 239, 207 and 141 interactions respectively (Table 1). Both the network topology and the set of regulatory logic functions used to construct the dynamical models have been included as supplementary information (S1 Table). References for added interactions from the ResNet mammalian database were additionally included in S1 Table.

**Table 1:** Applied case networks.

	Number of nodes	Number of interactions in the prior knowledge network
EMT	6	19
Th1-Th2	36	238
iPSC	52	207
hESC-cardiomyocyte	82	139

Three different examples were selected to validate PRUNET and illustrate its scalability and general applicability. The number of nodes and interactions for each applied example are included in the table.

The training sets used for the contextualization were taken from the same publications as the original GRNs [42–44] and have also been included in the supplements (S1 Table). Active and inactive genes were encoded as ‘1’ and ‘0’, respectively, in a Booleanized representation of the corresponding phenotypes used. It is worth noting here that the current implementation of PRUNET it is restricted to a training set consisting on the Booleanized representation of the initial and final state of a biological process. Future development may include multiple steady states and transient states. For the latter case, time series data would be required.

### Generating random subnetworks for validation of the algorithm

In order to validate the algorithm we compared for each included example the output of PRUNET against a population of subnetworks. To generate this population of random subnetworks we ran PRUNET without sampling the probability distribution of edges when generating a new population of subnetworks during the optimization process, but scoring and transferring the best subnetworks to the next population of subnetworks (elitism). This procedure mimics the random search of a subnetwork to explain the expression data for a total number of evaluated subnetworks that equals the number of subnetworks evaluated by PRUNET across the total number of iterations. Consequently, the resulting population of networks compared against those obtained after the regular application of PRUNET (sampling the probability distribution of edges) constitutes a fair comparison and shows the contribution of PRUNET with respect to a random search.

### General architecture

PRUNET is a cross platform tool with a graphical GUI developed entirely in Perl. The source code and executables for Windows, Mac OS and Linux are available online at http://prunet.sourceforge.net/ with versions for both 32 and 64 bit architecture.

### Usage and parameters

**Fig 2:**
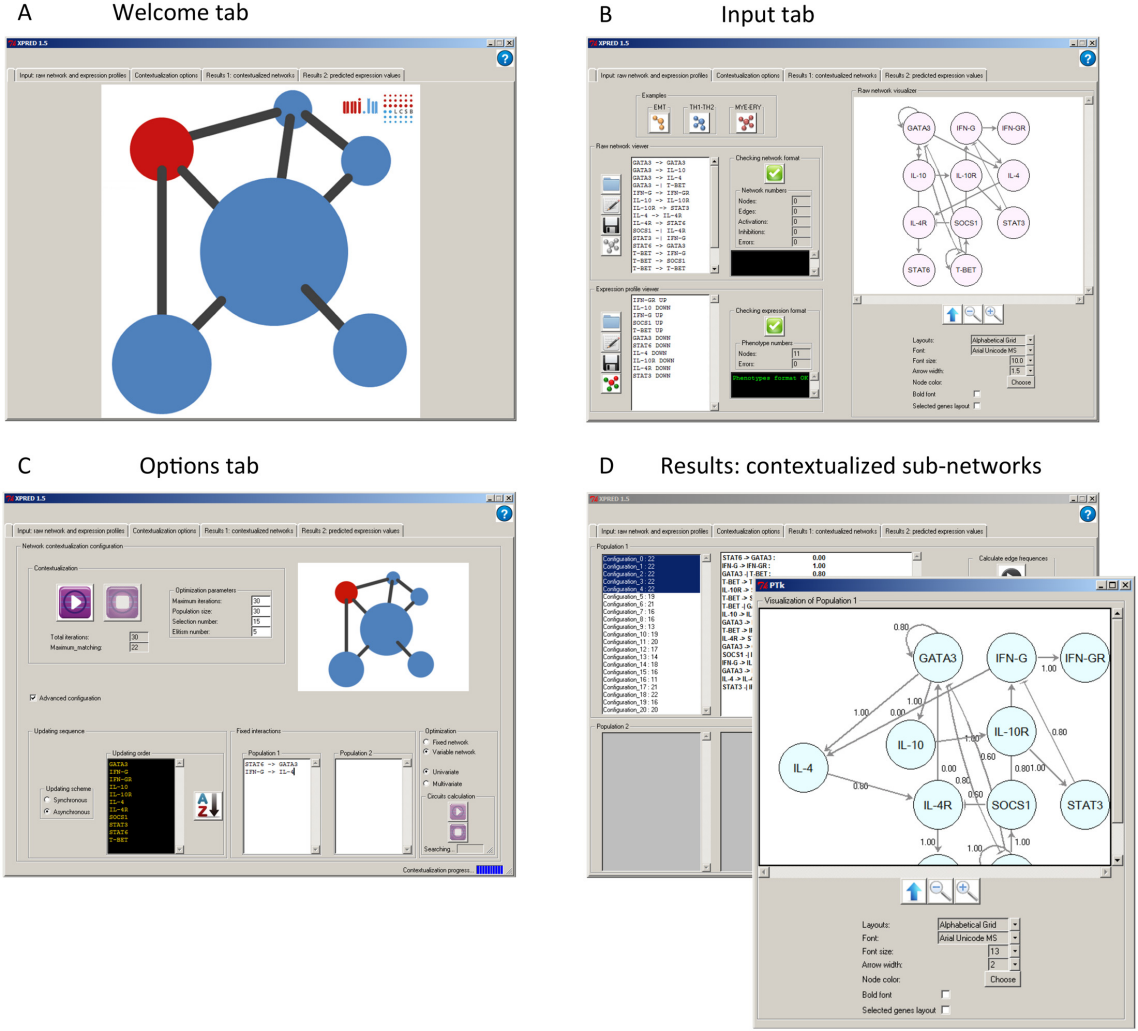
PRUNET interface. The graphical user interface is organized in tabs, namely a welcome tab (**A**), input tab (**B**), options tab (**C**) and results tab (D). Both the input and results tab include a network visualizer to facilitate the interaction of the user with the program.

The GUI is organized into five tabs (see Fig 2): i) welcome, ii) input, iii) options, iv) results 1: contextualized networks, and v) results 2: predicted expression values. Users can intuitively follow the order from left to right to use the application.

The input information can be copied and pasted into the corresponding text-box or loaded when browsing the file system. The output can be visualized in PRUNET or saved as files.

### Basic configuration parameters

#### Maximum iterations

This parameter refers to the maximum number of times the algorithm is going to be recursively applied or, in evolutionary terms, the number of subnetwork generations that are going to be sampled, scored and selected in order to yield better networks. It is the maximum because, despite users being able to stop the optimization process at anytime and collect ‘partial’ results, the program keeps working until it reaches this iteration number. In general, a bigger population size requires a higher number of iterations to obtain convergence (a final population of similar subnetworks).

#### Population size

This parameter refers to the number of subnetworks generated in each iteration. A high population size decreases the probability of a local optimum being reached but increases the computation time.

#### Selection number

This parameter refers to the number of top-scored subnetworks selected in each iteration. If the selection number is low the convergence to a population of similar subnetworks is quicker, but the optimization process could be slowed down due to the lack of variability.

#### Elitism number

This parameter refers to the number of the best historical subnetworks (in all iterations) that are directly transferred from one generation to the next to prevent the loss of best scoring subnetworks. We suggest using an elitism number not higher than half of the selection number in order to provide an optimization process with a degree of freedom.

### Advanced configuration parameters

Advanced configuration parameters can be set up in the tab ‘options’ when the corresponding radio-button is active. A description of these parameters is included on the website http://prunet.sourceforge.net/.

### Hardware used in the software comparison

For the comparison of PRUNET with CellNOptR, SigNetTrainer and XPRED we used a MacBook Pro with a processor of 2.4 GHz Intel Core i5 and memory of 8GB 1600 MHz DDR3.

## Results and Discussion

In order to validate and illustrate the usage of PRUNET, we used four different biological examples, namely EMT, Th1-Th2, iPSC and hESC-cardiomyocyte. For each of these four applied examples we performed two different tests: the prediction of missing expression values and the network response under specific perturbations.

Firstly, for the missing expression values prediction we performed a cross-validation analysis with repeated random sub-sampling validation; in other words, the datasets with the phenotypes expression profiles were randomly split into training and validation sets. The motivation behind this procedure was to evaluate how well networks were optimized using different training sets to predict known phenotypes.

We ran PRUNET ten times for each example and for three different training set sizes, comparing the predictions from the resulting population of optimized networks (N = 300) with predictions from a population of randomly generated subnetworks derived from the PKNs (see methods).

In all cases we applied the same configuration for the optimization, with a population size of 30, a selection number of 10 and 100 iterations of the algorithm. A T-test (with Welch’s correction) statistically supported the separation between the population of randomly generated subnetworks and the population of optimized networks, with a p-value < 0.01 for all the examples and training sizes (see S2 Table for detailed results).

**Fig 3:**
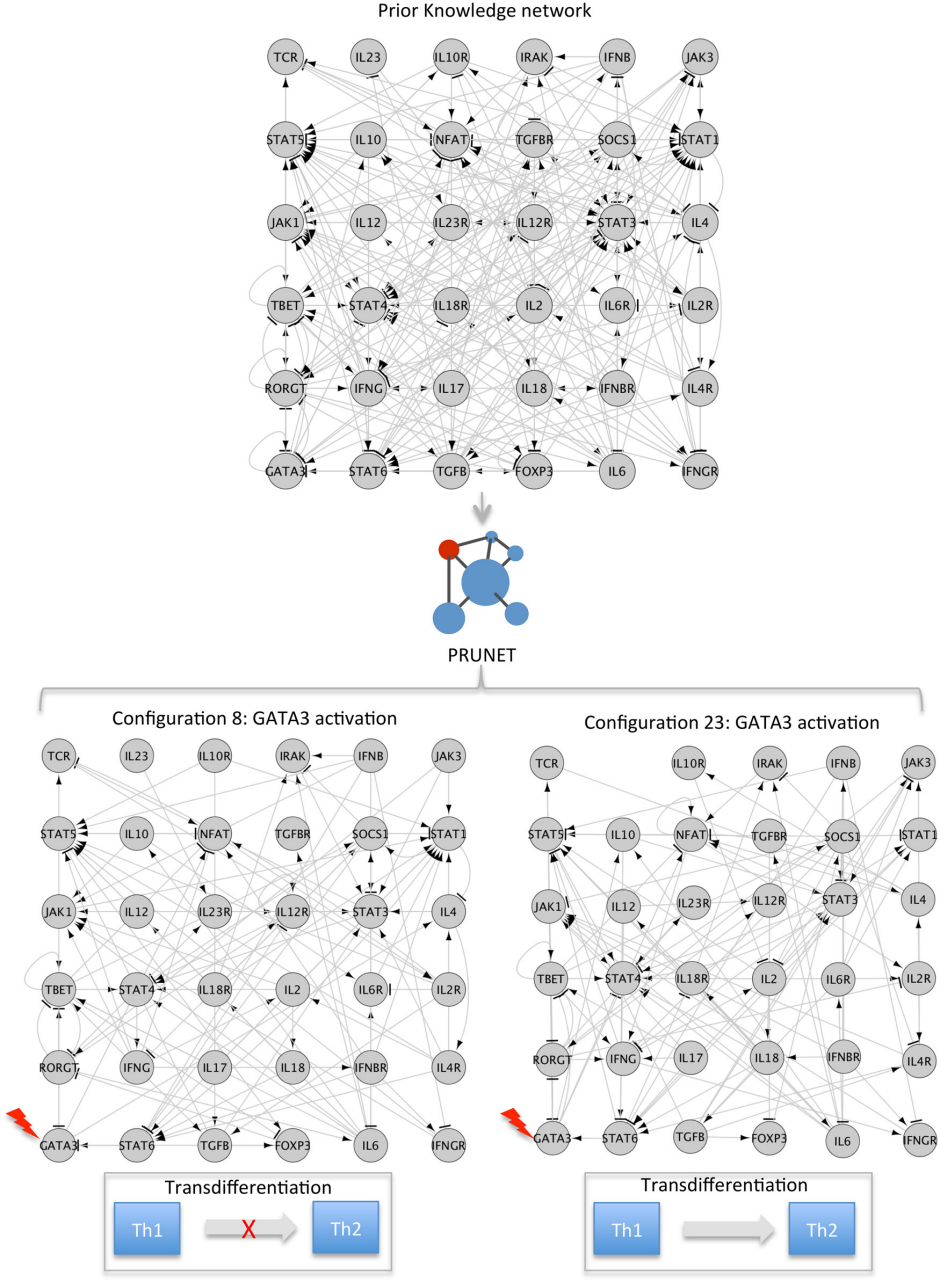
Simulated perturbations on alternative contextualized network may predict different response. In the case of Th1-Th2, 80% of the contextualized subnetworks, optimized to describe phenotypes Th1 and Th2 also showed the expected response during the simulated activation of GATA3, with the transition from Th1 to Th2 phenotypes. The rest of the subnetworks (20%) either remained in the same attractor (phenotype) or were transited to another that was different from the expected one (Th2).

Secondly, in order to assess the suitability of the optimized networks for predicting the dynamical behaviour of the model under well-characterized perturbations, we performed a simulation of such perturbations on the best five networks of each run (optimized using the complete phenotype to train the model) and compared the results obtained from these 50 networks with the expected response. Fig 3 shows an example on the Th1-Th2 case illustrating how the simulated perturbation (activation) of GATA3 was able to induce the transition from Th1 to Th2 in the contextualized network named as Configuration 23 but not in Configuration 8, where the system resulted in a cyclic attractor instead. The results for each applied example are described below.

Given that PRUNET is sensitive to the Booleanized phenotypes used for training the model, we have performed a sensitivity analysis for each biological example. In this analysis we have considered two situations: missing and wrong information about each single gene, and combinations of randomly-selected genes of different number. PRUNET generally demonstrated high tolerance for both missing and wrong information in all of the examples. The complete analysis is included in the supplements (S4 Table).

Finally, we compared PRUNET with other available software, namely CellNOptR[25], SigNetTrainer [30] and XPRED [23] (see Table 2), using four biological examples. The results showed that for the smallest example (EMT) CellNOptR and SigNetTrainer were faster than PRUNET (4 s, 0.47 s and 5.6 s, respectively), whereas XPRED was slower (2 m 30 s). All the tools obtained a perfect score (1). This score refers to the match between predicted and real phenotypes; a score of 1 represents a full agreement, while a score of 0.5 reflects a correct description of 50% of the genes for all the attractors. However, PRUNET displayed a better scalability for larger examples. CellNOptR was not able to run either the Th1-Th2 or the iPSC or the hESC-cardiomyocyte examples. SigNetTrainer obtained a worse score than PRUNET in the Th1-Th2 example (0.51 and 0.88, respectively) despite the fact that it was faster (3.7 s and 7 m 25 s, respectively), and it was not able to run the iPSC and hESC-cardiomyocyte examples. With respect to PRUNET, XPRED obtained similar scores for the four examples, although its computation time was much longer (see Table 2).

**Table 2:** Comparison of PRUNET with similar available software.

	CellNOptR	SigNetTrainer	XPRED	PRUNET
**EMT**	Computation time: **0 h 0 m 4 s** Score = 1	Computation time: **0 h 0 m 0,47 s** Score = 1	Computation time: **0 h 2 m 30 s** Score = 1	Computation time: **0 h 0 m 5,6 s** Score = 1
**Th1-Th2**		Computation time: **0 h 0 m 3,7 s** Score = 0,51	Computation time: **176 h 47 m 34 s** Score = 0,90	Computation time: **0 h 7 m 25 s** Score = 0,88
**iPSC**			Computation time: **63 h 33 m 45 s** Score = 0,94	Computation time: **0 h 2 m 17 s** Score = 0,95
**hESC-cardiomyocytes**			Computation time: **17 h 30 m 0 s** Score = 0,83	Computation time: **0 h 1 m 37 s** Score = 0,82

Scores represent the match between predicted and real phenotypes; a score of 1 represents a full agreement between training set and model predictions, while a score of 0.5 reflects a correct description of 50% of the training set.

### Applied example I: EMT

A transient phenomenon called epithelial to mesenchymal transition (EMT) occurs during both regular embryonic development and as a part of the metastatic cascade initiated by the breakdown of epithelial cell homeostasis in carcinomas. During the EMT, cells change their transcriptional programs, which leads to phenotypic and functional alterations, including the loss of epithelial features like cell-cell adhesions and cell polarity and the gain of cell motility and mesenchymal and stem-like properties. EMT can be initiated by multiple pathways converging on the activation of EMT inducers. The EMT example consists of a regulatory core, with six genes and eighteen interactions. To contextualize the PKN we used the Booleanized representation of the epithelial and mesenchymal phenotypes.

**Fig 4:**
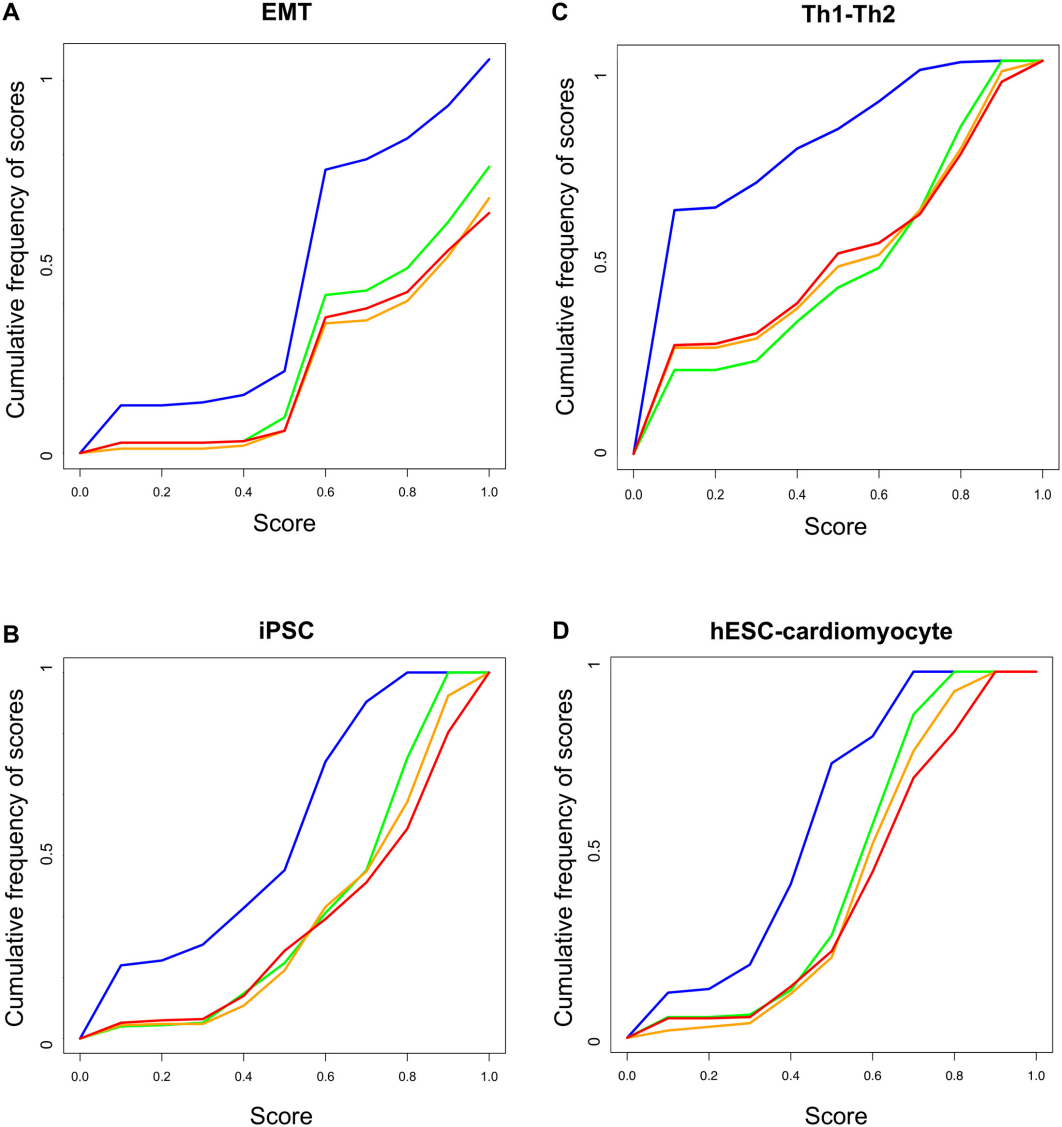
Validation of PRUNET using four biological examples. The scores obtained after the application of PRUNET to four biological examples, namely epithelial to mesenchymal transition (EMT), Th1-Th2 transdifferentiation (Th1-Th2), induced pluripotent stem cells (iPSC) and cardiomyocyte differention of human embryonic stem cells (hESC-cardiomyocyte), were compared with the scores obtained by a population of randomly generated subnetworks from the prior knowledge network. Green, orange and red lines represent the cumulative frequencies of scores obtained with training sets of a half, an intermediate number (different for each case) and all of the genes respectively, whereas the blue line represents the scores obtained from the population of subnetworks randomly generated from the prior knowledge network. The separation between blue and the other lines represents the contribution of PRUNET to the prior knowledge network in terms of describing the known phenotypes of different training set sizes.

For the expression values prediction, we used ten randomly selected training sets of three, four and six genes. Fig 4 A shows the cumulative frequencies of the PRUNET’ scores and shows a clear separation between the populations of randomly generated subnetworks (blue line) and optimized networks (green, orange and red lines for three, four and six genes in the training set respectively). The mean of the scores (ranging from 0 to 1) of the different populations were 0.58, 0.76, 0.80 and 0.79 for the randomly generated subnetworks and subnetworks trained with three, four and six genes respectively.

The EMT network includes SNAI1, a triggering factor for EMT, which has been validated by experimental perturbation of this gene [42]; the activation of SNAI1 was simulated and evaluated for this example on the five best subnetwork from each run (fifty subnetworks in total). In this particular case, 20% (N = 12) of the subnetworks showed the expected response, with a transition from epithelial to mesenchymal phenotypes and a perfect match with the latter phenotype. The remaining networks either stayed in the epithelial phenotype or became in cyclic attractors.

### Applied example II: Th1-Th2

T lymphocytes are immune cells that can be roughly classified in two main categories: T-helper and T-cytotoxic. T-helper cells take part in cell- and antibody-mediated immune responses and they are sub-divided in Th0 (precursor), effector Th1, Th2, Th17 and Treg. To contextualize the PKN, including thirty-six genes and two hundred and thirty-nine interactions we focused on the cellular transition between Th1 and Th2, using the Booleanized representation of Th1 and Th2 phenotypes in the optimization process.

For the expression values prediction, we used ten randomly selected training sets of eighteen, twenty-seven and thirty-six genes. Fig 4B shows a clear separation between the populations of randomly generated subnetworks (blue line) and optimized networks (green, orange and red lines for eighteen, twenty-seven and thirty-six genes in the training set respectively). The mean of the scores of the different populations were 0.17, 0.48, 0.50 and 0.47 for randomly generated subnetworks and subnetworks trained with eighteen, twenty-seven and thirty-six genes respectively.

The perturbation of GATA3 has been reported as being capable of inducing the transdifferentiation from Th1 to Th2 [48]. We simulated and evaluated such a perturbation on the best five optimized subnetworks of each run (fifty subnetworks in total). In this case, 80% of the optimized subnetworks (N = 40) showed a transition to a new attractor with a match with the Th2 phenotype of 75% or more.

### Applied example III: iPSC

Somatic cells can be converted back to the stem cell states (the so called induced pluripotent stem cells or iPSC) by overexpressing three to four genes in the somatic cells essential for regulating pluripotency. We took a PKN, with 52 genes and 207 interactions, based on the GRN involved in regulating pluripotency and hESC differentiation proposed by Chang et al. [49] and used a Booleanized representation of the phenotypes corresponding to ‘differentiated’ and ‘pluripotent’ to train the model.

Once again, for the expression values prediction, we used ten randomly generated traning sets of 26, 39 and 52 genes.

Fig 4C shows a separation between the populations of randomly generated subnetworks (blue line) and optimized networks (green, orange and red lines for 26, 39 and 52 genes in the training set respectively). The mean of the scores of the different populations were 0.42, 0.64, 0.66 and 0.67 for randomly generated subnetworks and the subnetworks trained with twenty-six, thirty-nine and fifty-two genes respectively.

The combined perturbation of POU5F1, SOX2 and KLF4 is known to be capable of inducing pluripotent cellular states [50]. We simulated the activation of these genes on the best 50 optimized subnetworks from the iPSC example to evaluate the transition from ‘differentiated’ to ‘pluripotent’ phenotypes. According to our simulations, 32% (N = 16) of the optimized subnetworks showed a transition to an attractor with at least 75% of matching with the expected ‘pluripotent’ phenotype. The rest of the networks remained in the same initial attractor (‘differentiated’) or became a new one that has lower matching with the expected phenotype.

### Applied example IV: hESC-cardiomyocyte

Human embryonic stem cells (hESCs) can be differentiated *in vitro* into cells of different lineages following specific cell culture protocols. Recently, several protocols have been developed to differentiate hESCs into cardiomyocytes [51–54], thereby providing an ideal *in vitro* model to study heart development and disease. Gu and co-workers constructed a GRN from literature connecting differentially expressed genes between hESC and cardiomyocytes differentiated by perturbation of the WNT pathway [54]. We contextualized the original raw GRN using PRUNET and challenged the top five optimized subnetworks of ten different runs of the algorithm to reproduce the cellular transition from hESCs to cardiomyocyte triggered by the inhibition of SOX2 and activation of MEF2C resulting from the inhibition of the WNT pathway. The network was contextualized using the complete expression profile of hESCs and cardiomyocytes (83 genes) and 10 randomly-selected training sets of 42 and 63 genes. Fig 4D shows the separation between the scores of randomly-generated subnetworks and subnetworks obtained using Prunet for training sets of 42, 63 and 83 genes. The mean of the scores for the population of random subnetworks was 0.39, whereas for training sets of 42, 63 and 83 genes, the mean scores were 0.54, 0.58 and 0.59, respectively.

Concerning the combined perturbation of SOX2 (inhibited) and MEF2C (activated), our simulations showed that 36% (N = 18) of the best subnetworks exhibited a transition to an attractor with at least 50% matching with the expected ‘cardiomyocyte’ phenotype. The rest of the networks remained in the same initial attractor (‘hESC’) or reached another attractor by matching with the ‘cardiomyocyte’ phenotype lower than 50% of the genes.

It is worth noting here that in the four examples, although the population of 50 best subnetworks selected to simulate the perturbation experiments nicely explained the expected phenotypes, a variable fraction of these subnetworks failed to explain the expected response (80%, 20%, 66% and 64% for EMT, Th1-Th2, iPSC and hESC-cardiomyocyte respectively). An exploration of the network topology within the population of the top fifty subnetworks revealed that the diversity in network topology seems to be associated with worse performance in accurate prediction response to perturbations. In other words, diverse alternative subnetworks used to explain the same attractors can also cause diverse responses under perturbation. This observation suggests that the convergence to similar or identical subnetworks when running PRUNET multiple times (the algorithm is heuristic) could be used as an indicator of the reliability of the predicted response to perturbations. This concept requires, of course, further investigation and development.

It is also worth mentioning here that only the network topology and not the logic functions are optimized by PRUNET. An inhibitory dominant system is assumed by default (see methods); however, alternative regulatory systems are partially compensated by the optimization. For example, let us suppose that a given gene is inhibited by a second one and activated by a third one. If all genes are active in one of the phenotypes, PRUNET will remove the inhibition because it assumes that if an inhibitor is active its effect is dominant, and in the adopted Boolean formalism that implies that the regulated gene should be inactive. This means that the network is not only contextualized to the biological context but also to the adopted set of regulatory logic rules. Despite both the removal of dominant interactions inconsistent with the state of their targets and changing the regulatory rules that can equally explain the observed phenotypes, the predicted response to perturbations may be different; it is not equivalent that an inhibitory effect is not dominant in a particular case (a given steady state) and that an inhibitory effect never happens. However, we have shown that a fraction of the resulting contextualized networks were able to correctly predict the response to specific perturbations in spite of the regulatory logic assumed by default in the biological examples included in this work.

If different regulatory rules are known they can be encoded by the addition of artificial nodes in the network, as proposed by Garg et al. [18]. Looking for optimal set of rules constitutes a natural expansion of the present implementation of PRUNET.

Other future developments may include the implementation of the same network contextualization strategy for a discrete system not Boolean, which should allow de the gene activity and network dynamics to be described more precisely, and the optimization algorithm to be adapted to work with cyclic attractors in order to make PRUNET suitable for contextualizing the underlying network of biological processes with oscillatory behaviour.

## Conclusion

The last years have witnessed an increase in the size and complexity of PKN, partly due to automated network creation techniques exploiting information from literature. These techniques tend to be inclusive rather than exclusive in an attempt to capture all relevant biological events. The resulting networks usually merge a number of context dependent interactions. This is partly due to the presence of such interactions as frequently these networks cannot be used directly to simulate biological processes under specific biological contexts, defined by features like the cell type, tissue or experimental conditions.

PRUNET was conceived precisely to identify and remove such interactions in an attempt to adapt a general PKN to describe biological processes in specific conditions. The resulting contextualized network(s) constitute an optimized model able to describe known phenotypes and can ben used to simulate transitions between them in response to perturbations. PRUNET assumes that the initial PKN is complete enough to describe the biological process under study; it is beyond its scope to address the problem of network incompleteness.

The application of PRUNET provides an added value to PKN and can be useful in assisting with the hypothesis generation and guiding researchers in the experiment design in biological and medical research.

## Availability and Requirements

### Home page

The software, examples and tutorial are accessible through the website at http://prunet.sourceforge.net/

### Operating system

Windows (32 and 64 bit), Mac OS and Linux (32 and 64 bit).

### Requirements

In order to be able to run the Perl program ActivePerl 5.14 or later and the Perl package, Tk-804.027 should be installed. In addition, running the source code in Mac OS also requires the installation of X11.app.

### License

PRUNET is free for academic purposes but requires a license from the authors for any commercial purposes. The software is available without user registration.

#### Further information

A quick start user’s guide and an explanation about advanced configuration are included on the application homepage.

## Supporting Information

S1 Fig Sensitivity analysis EMT. Boxplots summarize the distribution of scores of contextualized networks when either missing (in green) or wrong (orange) information is given to PRUNET. **A) Single genes.** This analysis refers to missing or wrong information about specific genes. The results indicated that SNAI1 was the most sensitive gene of the model; **B) Combinations of genes.** This analysis refers to missing or wrong information about combinations of genes randomly selected.
(TIFF)

S2 Fig Sensitivity analysis Th1-Th2 single genes. The boxplot summarizes the distribution of scores of contextualized networks when either missing (in green) or wrong (orange) information about specific genes is given to PRUNET. The results indicated that FOXP3, IL18R and TBET were the most sensitive gene of the model.
(TIFF)

S3 Fig Sensitivity analysis Th1-Th2 combinations genes. The boxplot summarizes the distribution of scores of contextualized networks when either missing (in green) or wrong (orange) information about combinations randomly selected is given to PRUNET.
(TIFF)

S4 Fig Sensitivity analysis iPSC single genes. The boxplot summarizes the distribution of scores of contextualized networks when either missing (in green) or wrong (orange) information about specific genes is given to PRUNET. The results indicated that NANOG the most sensitive gene of the model.
(TIFF)

S5 Fig Sensitivity analysis iPSC combinations of genes. The boxplot summarizes the distribution of scores of contextualized networks when either missing (in green) or wrong (orange) information about combinations randomly selected is given to PRUNET.
(TIFF)

S6 Fig Sensitivity analysis hESC-cardiomyocyte single genes. The boxplot summarize the distribution of scores of contextualized networks when either missing (in green) or wrong (orange) information about specific genes is given to PRUNET.The results indicated that POU5F1 was the most sensitive gene of the model.
(TIFF)

S7 Fig Sensitivity analysis hESC-cardiomyocyte combinations of genes. The boxplot summarize the distribution of scores of contextualized networks when either missing (in green) or wrong (orange) information about combinations randomly selected is given to PRUNET.
(TIFF)

S1 Table Prior knowledge networks, Boolean models and Booleanized phenotypes (training set). (XLSX)

S2 Table PRUNET validation results. (XLSX)

S3 Table Simulated perturbation results. (XLSX)

S4 Table Sensitivity analysis results. (XLSX)

S1 Text Sensitivity analysis of networks contextualized with PRUNET in four applied cases: EMT, Th1-Th2, iPSC and hESC-cardiomyocyte. (DOC)
