# Supplementary figures and images for: Discrete Logic Modelling Optimization to Contextualize Prior Knowledge Networks Using PRUNET
Source: PLoS One. 2015 Jun 9;10(6):e0127216. doi: 10.1371/journal.pone.0127216 (PMC4461287; doi:10.1371/journal.pone.0127216)

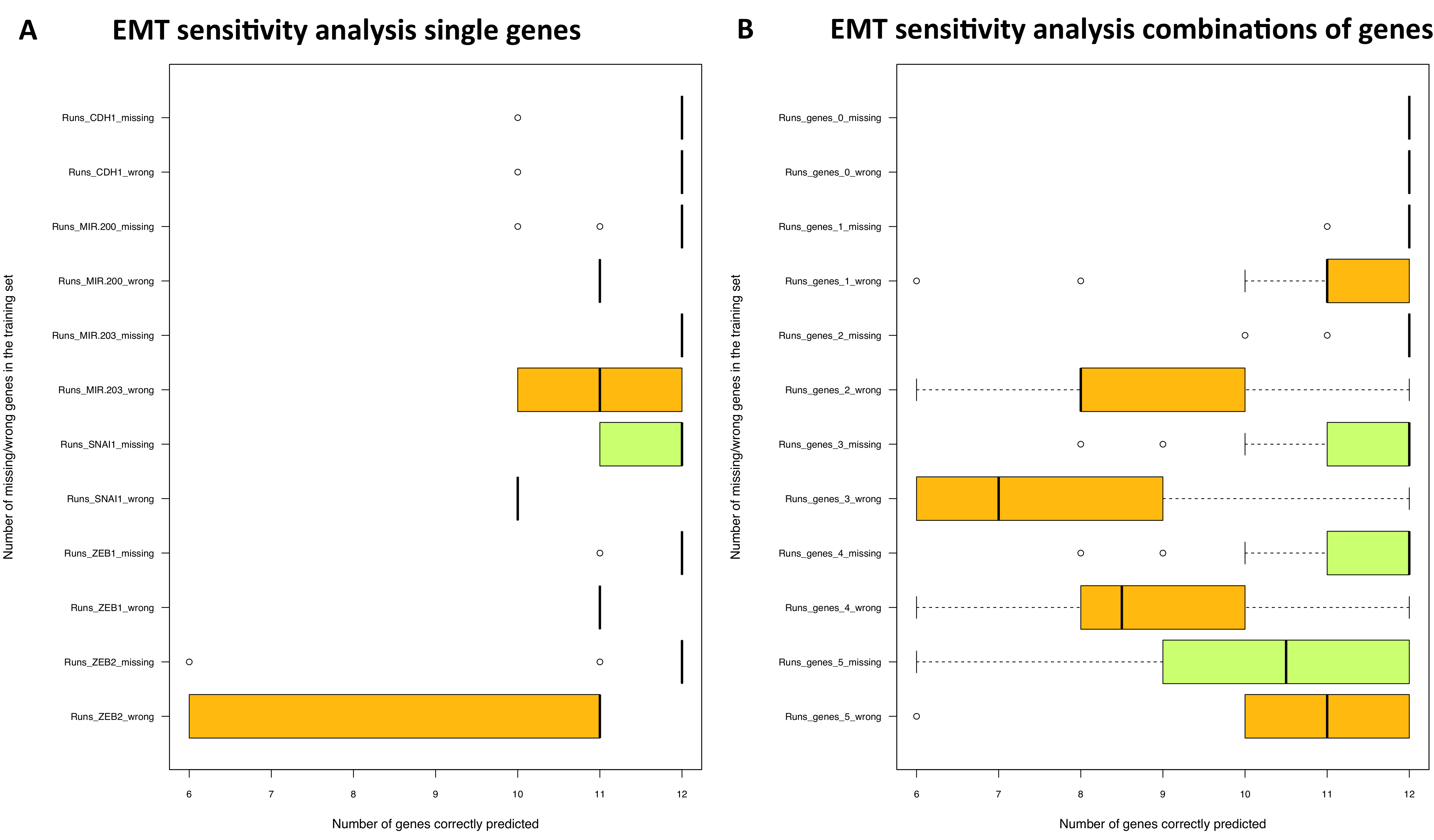

Supplement: S1 Fig — Boxplots summarize the distribution of scores of contextualized networks when either missing (in green) or wrong (orange) information is given to PRUNET. A) Single genes. This analysis refers to missing or wrong information about specific genes. The results indicated that SNAI1 was the most sensitive gene of the model; B) Combinations of genes. This analysis refers to missing or wrong information about combinations of genes randomly selected. (TIFF) [file pone.0127216.s001.tiff]

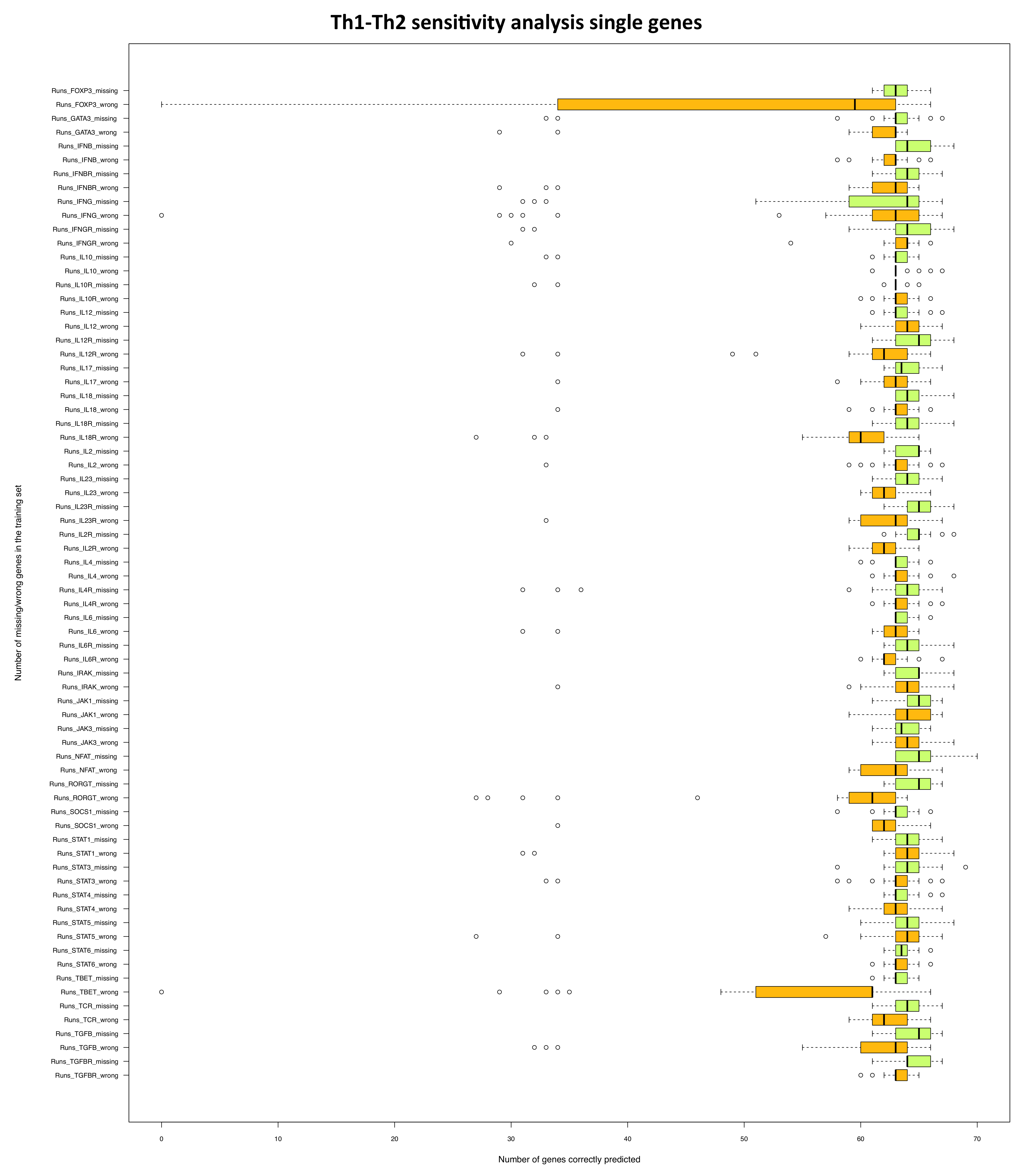

Supplement: S2 Fig — The boxplot summarizes the distribution of scores of contextualized networks when either missing (in green) or wrong (orange) information about specific genes is given to PRUNET. The results indicated that FOXP3, IL18R and TBET were the most sensitive gene of the model. (TIFF) [file pone.0127216.s002.tiff]

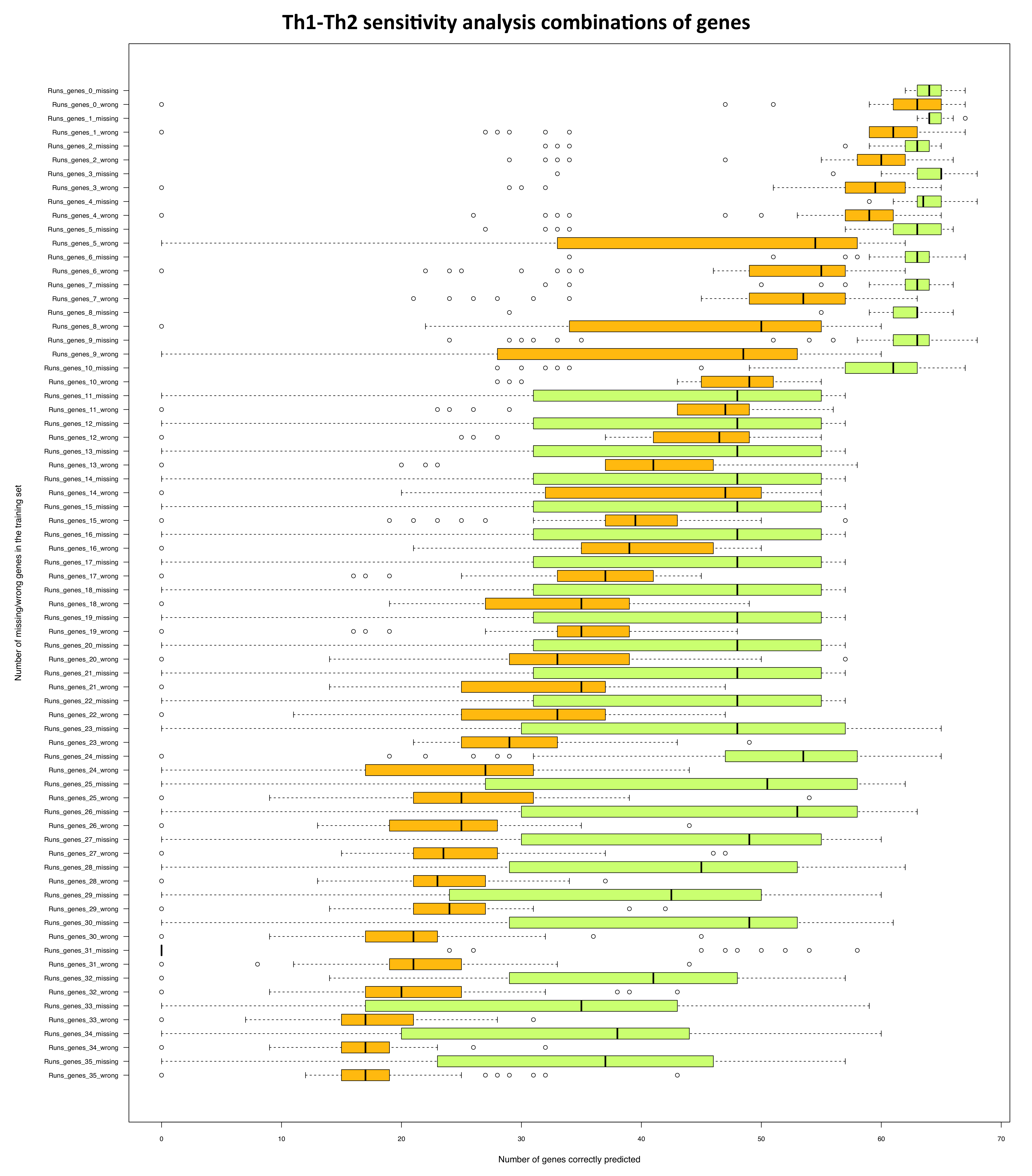

Supplement: S3 Fig — The boxplot summarizes the distribution of scores of contextualized networks when either missing (in green) or wrong (orange) information about combinations randomly selected is given to PRUNET. (TIFF) [file pone.0127216.s003.tiff]

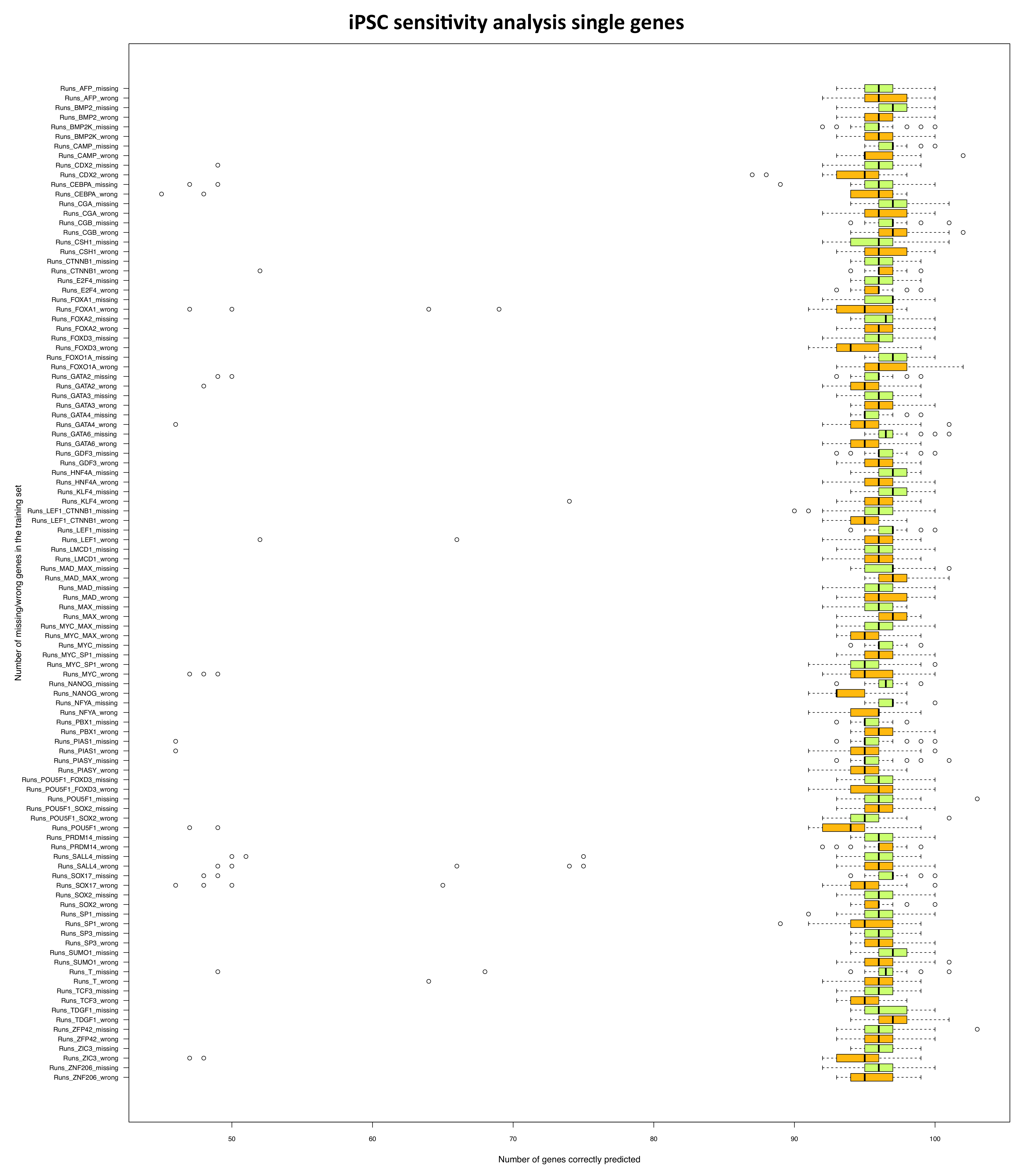

Supplement: S4 Fig — The boxplot summarizes the distribution of scores of contextualized networks when either missing (in green) or wrong (orange) information about specific genes is given to PRUNET. The results indicated that NANOG the most sensitive gene of the model. (TIFF) [file pone.0127216.s004.tiff]

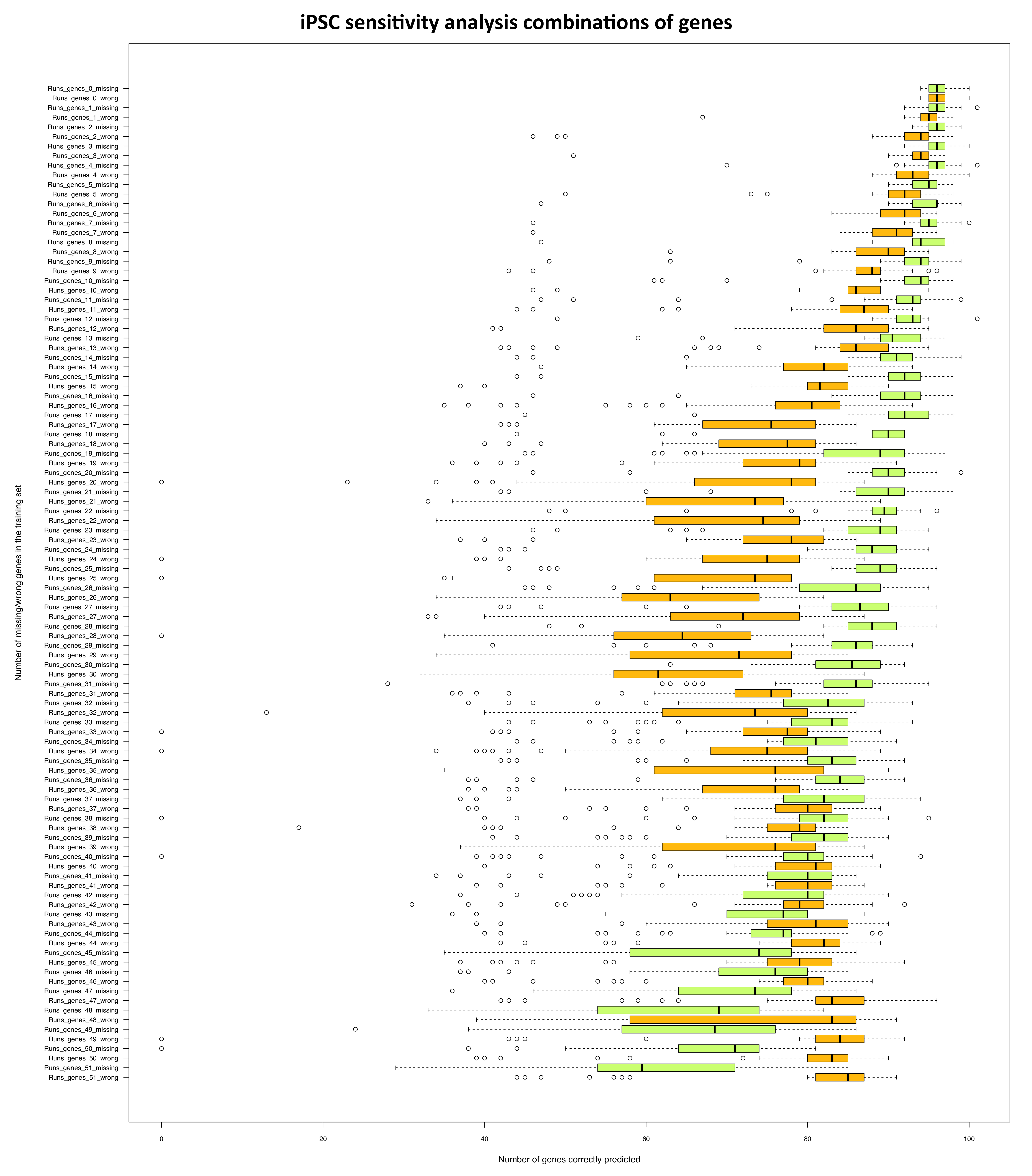

Supplement: S5 Fig — The boxplot summarizes the distribution of scores of contextualized networks when either missing (in green) or wrong (orange) information about combinations randomly selected is given to PRUNET. (TIFF) [file pone.0127216.s005.tiff]

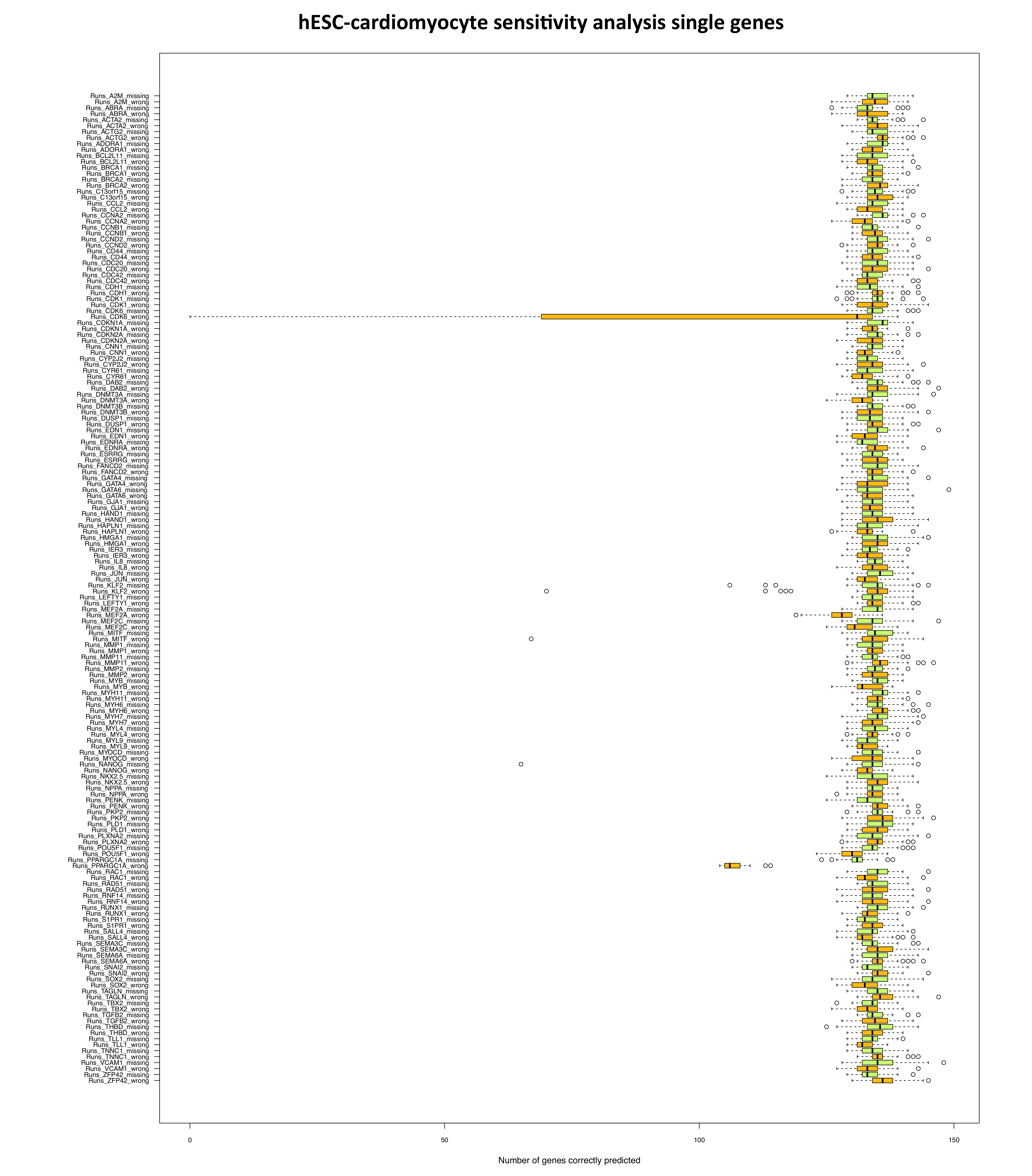

Supplement: S6 Fig — The boxplot summarize the distribution of scores of contextualized networks when either missing (in green) or wrong (orange) information about specific genes is given to PRUNET.The results indicated that POU5F1 was the most sensitive gene of the model. (TIFF) [file pone.0127216.s006.tiff]

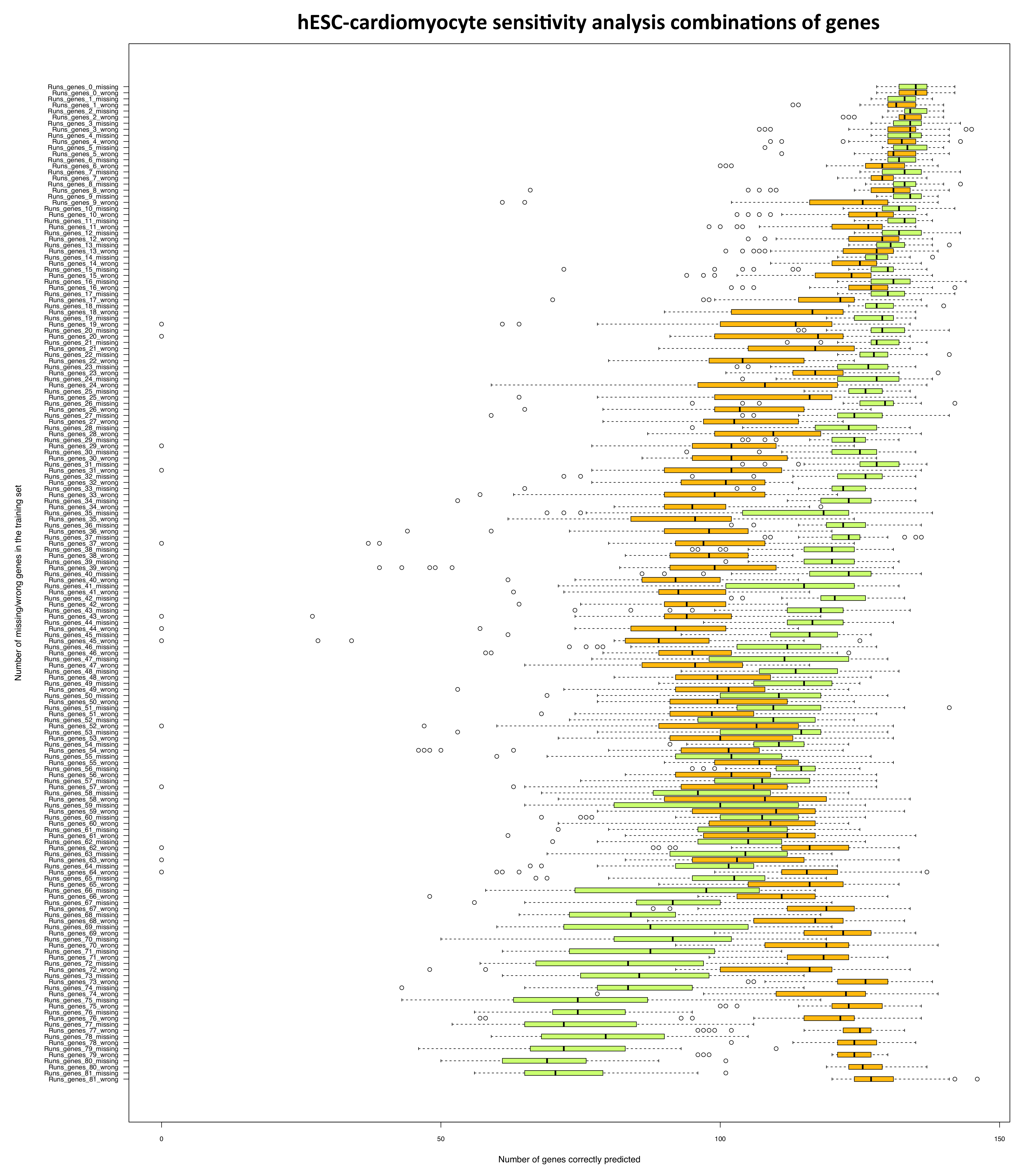

Supplement: S7 Fig — The boxplot summarize the distribution of scores of contextualized networks when either missing (in green) or wrong (orange) information about combinations randomly selected is given to PRUNET. (TIFF) [file pone.0127216.s007.tiff]
