# Supplementary material for: Discrete Logic Modelling Optimization to Contextualize Prior Knowledge Networks Using PRUNET
Source: PLoS One. 2015 Jun 9;10(6):e0127216. doi: 10.1371/journal.pone.0127216 (PMC4461287; doi:10.1371/journal.pone.0127216)
Supplement: S1 Text — (DOC) [file pone.0127216.s012.doc]

**Sensitivity analysis**

We performed a detailed sensitivity analysis in order to evaluate the effect of missing and wrong information in the Booleanized phenotypes that PRUNET takes as the input for each biological example included in this work, namely the epithelial to mesenchymal transition (EMT), T-Helper lymphocytes transdifferentiation (Th1-Th2), the induction of pluripotent stem cells (iPSC), and the differentiation of human embryonic stem cells (hESC) into cardiomyocytes (hESC-cardiomyocyte).

To this end, we performed two separate analyses for each example: a) sensitivity of independent genes; and b) sensitivity of combinations of genes that were randomly selected.

**Sensitivity of independent genes**

For each gene, we evaluated two situations: ‘missing’ and ‘wrong’ information. ‘Missing’ information means that the gene under study was removed from the training set, whereas ‘wrong’ information indicates that the correct value was flipped from its original Boolean state (either ‘0’ or ‘1’) to the opposite value. Under such conditions, PRUNET was applied 10 times with a population size of 30, selection number of 15, and 100 iterations of the algorithm. The top 5 networks of each run (50 in total) were collected and used to compare the effect across the genes and biological examples. It is important to note here that the scores used for the comparison were those obtained by the population of collected networks with the complete/correct training set. The results of the sensitivity analysis of independent genes are summarized in S1A, S2, S4 and S6 Figs.

**Sensitivity of combinations of randomly-selected genes**

For any number of genes from 1 to N-1 (N = total number of genes in the network), we randomly generated combinations of genes to be either removed from the training set or flipped to their opposite Boolean value in order to evaluate the effect of ‘missing’ and ‘wrong’ information, respectively. We applied PRUNET 10 times with the same parameters used for the sensitivity analysis of independent genes (see above). The top 5 networks of each run (50 in total) were evaluated using the complete and correct training set.

The results of the sensitivity analysis of combinations of genes are summarized in S1B, S3, S5 and S7 Figs.

**Results**

In general, PRUNET showed high tolerance for both ‘missing’ and ‘wrong’ information, performing reasonably well even with 50% of the training set. As expected, ‘wrong’ information was, in general, worse than ‘missing’ information. However, the analysis of combinations of genes demonstrated in all of the examples (except in Th1-Th2) that at some point (~50% of the training set) networks trained with ‘wrong’ information performed better than those with ‘missing’ information. This effect results because the more we change a given Booleanized phenotype, the more similar to the other Booleanized phenotype it becomes when the two phenotypes are complementary. This effect was not observed in the Th1-Th2 example due to the low complementarity between the two phenotypes. Of course, this situation never happens with ‘missing’ information.

The robustness against noisy information was expected, to some extent, due to the algorithm accepting mismatches in a few genes, provided that the global match between the computed attractors and Booleanized phenotypes is good. However, of course not all genes are equally informative, and the performance of the algorithm is affected to different extents depending on each particular case. The most sensitive genes for each biological example were:

- EMT: SNAI1 (see S1A Fig.)
- Th1-Th2: FOXP3, IL18R and TBET (see S2 Fig.)
- iPSC: NANOG (see S4 Fig)
- hESC-cardiomyocyte: POU5F1 (see S6 Fig.)

| 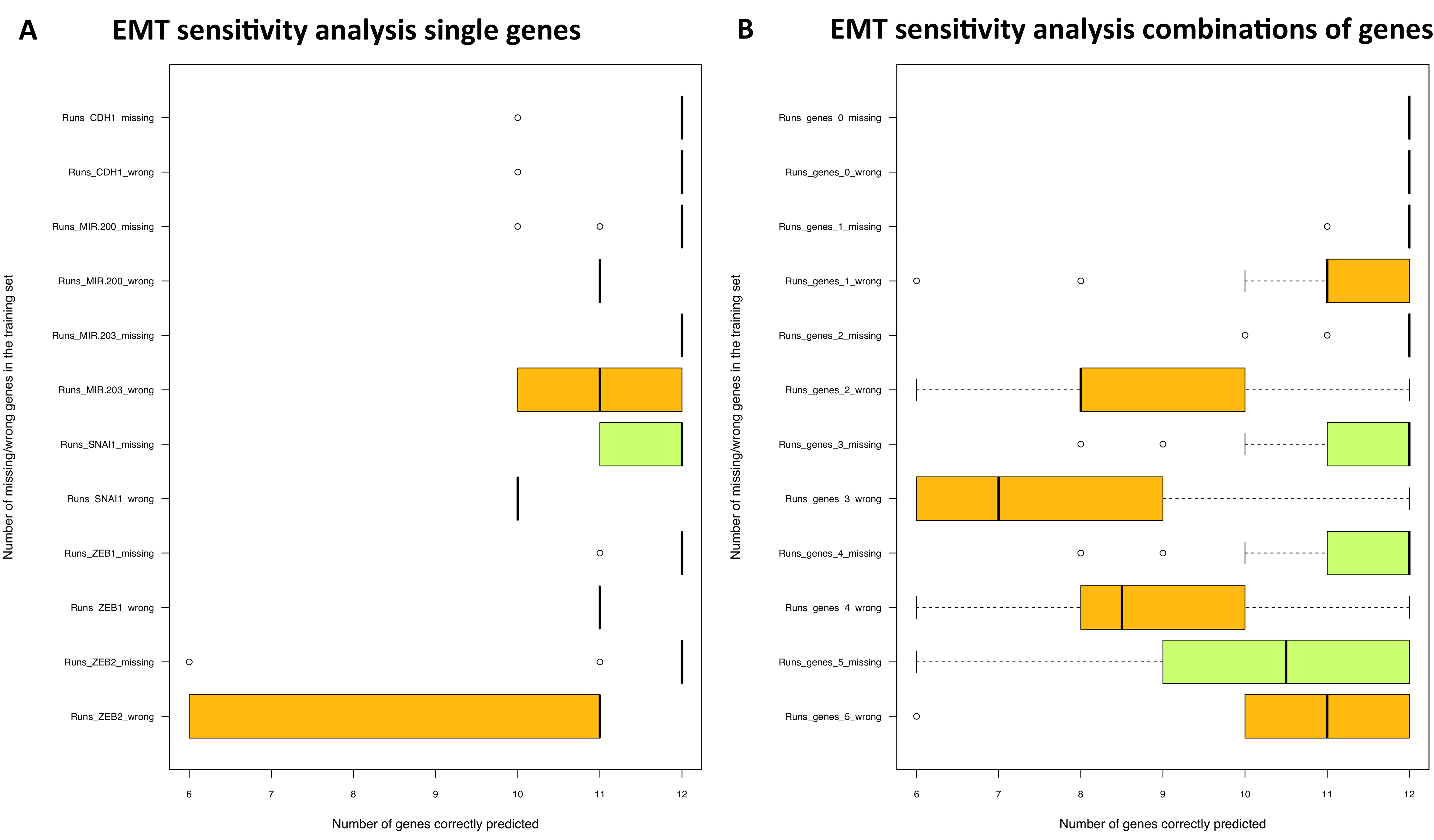 |
| --- |
| **S1 Fig. Sensitivity analysis EMT.** Boxplots summarize the distribution of scores of contextualized networks when either missing (in green) or wrong (orange) information is given to PRUNET. **A) Single genes.** This analysis refers to missing or wrong information about specific genes. The results indicated that SNAI1 was the most sensitive gene of the model; **B) Combinations of genes.** This analysis refers to missing or wrong information about combinations of genes randomly selected. |

|  |
| --- |
| **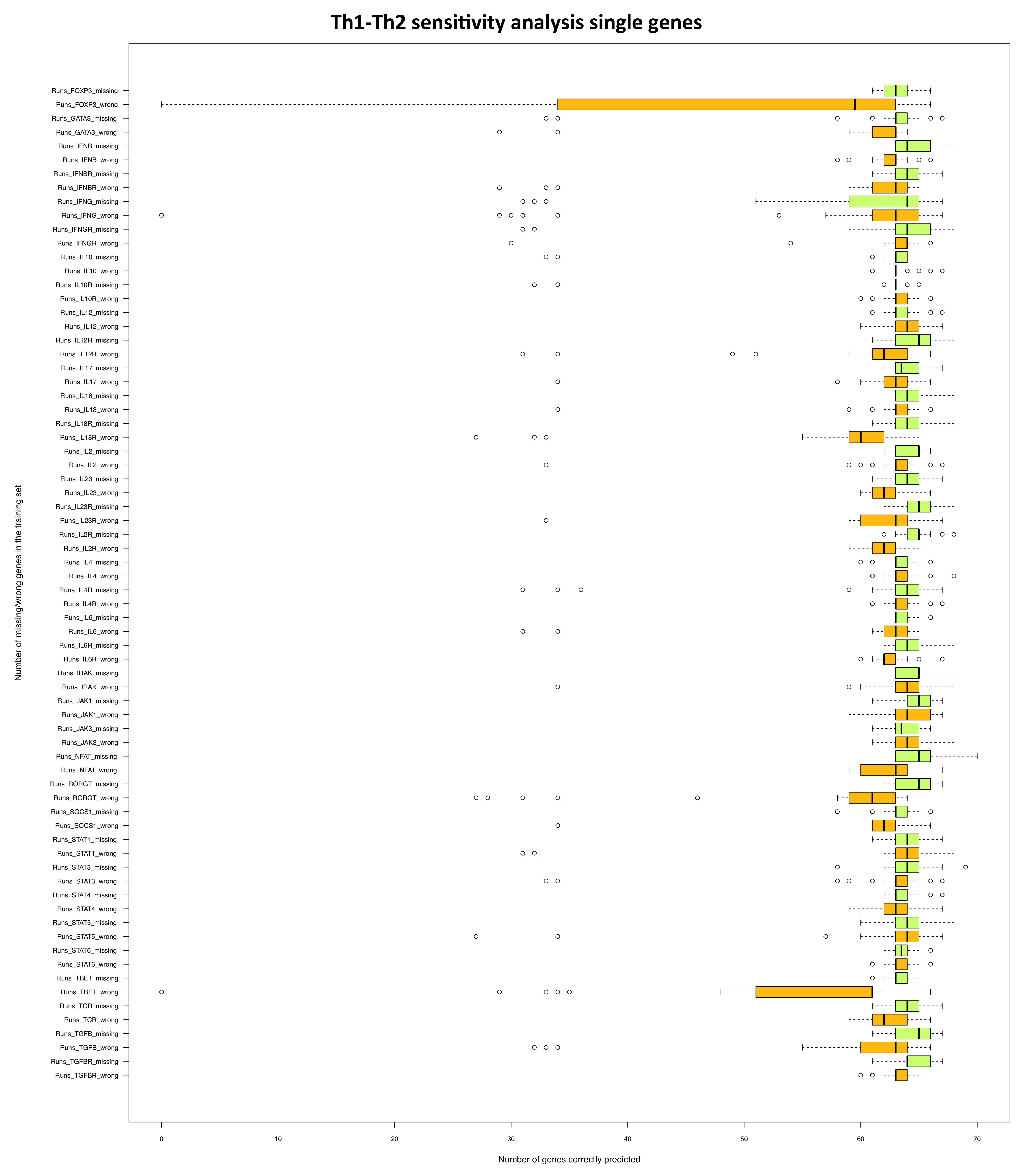**  **S2 Fig. Sensitivity analysis Th1-Th2 single genes.** The boxplot summarize the distribution of scores of contextualized networks when either missing (in green) or wrong (orange) information about specific genes is given to PRUNET. The results indicated that FOXP3, IL18R and TBET were the most sensitive gene of the model. |

| 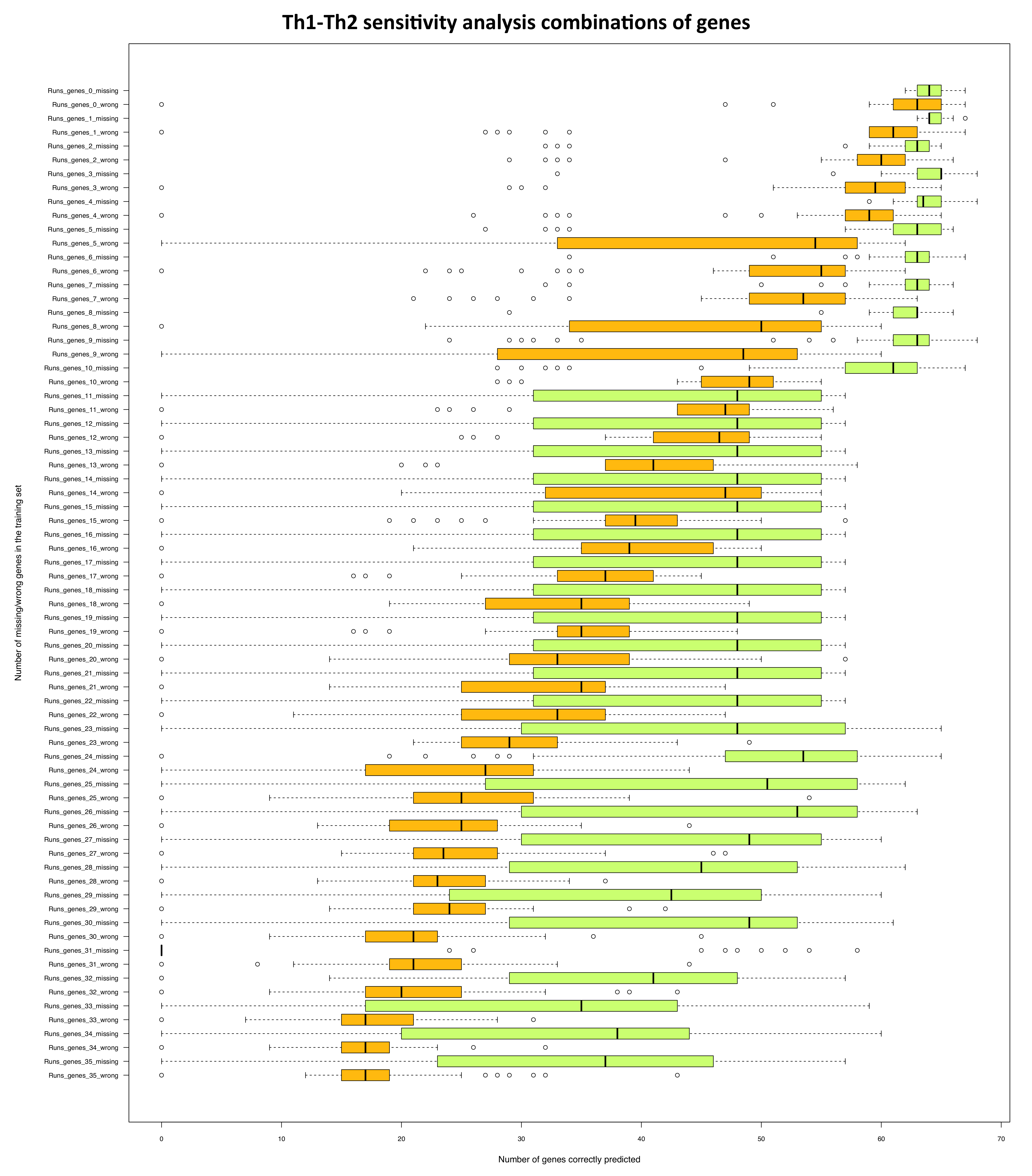 |
| --- |
| **S3 Fig. Sensitivity analysis Th1-Th2 combinations genes.** The boxplot summarize the distribution of scores of contextualized networks when either missing (in green) or wrong (orange) information about combinations randomly selected is given to PRUNET. |

| 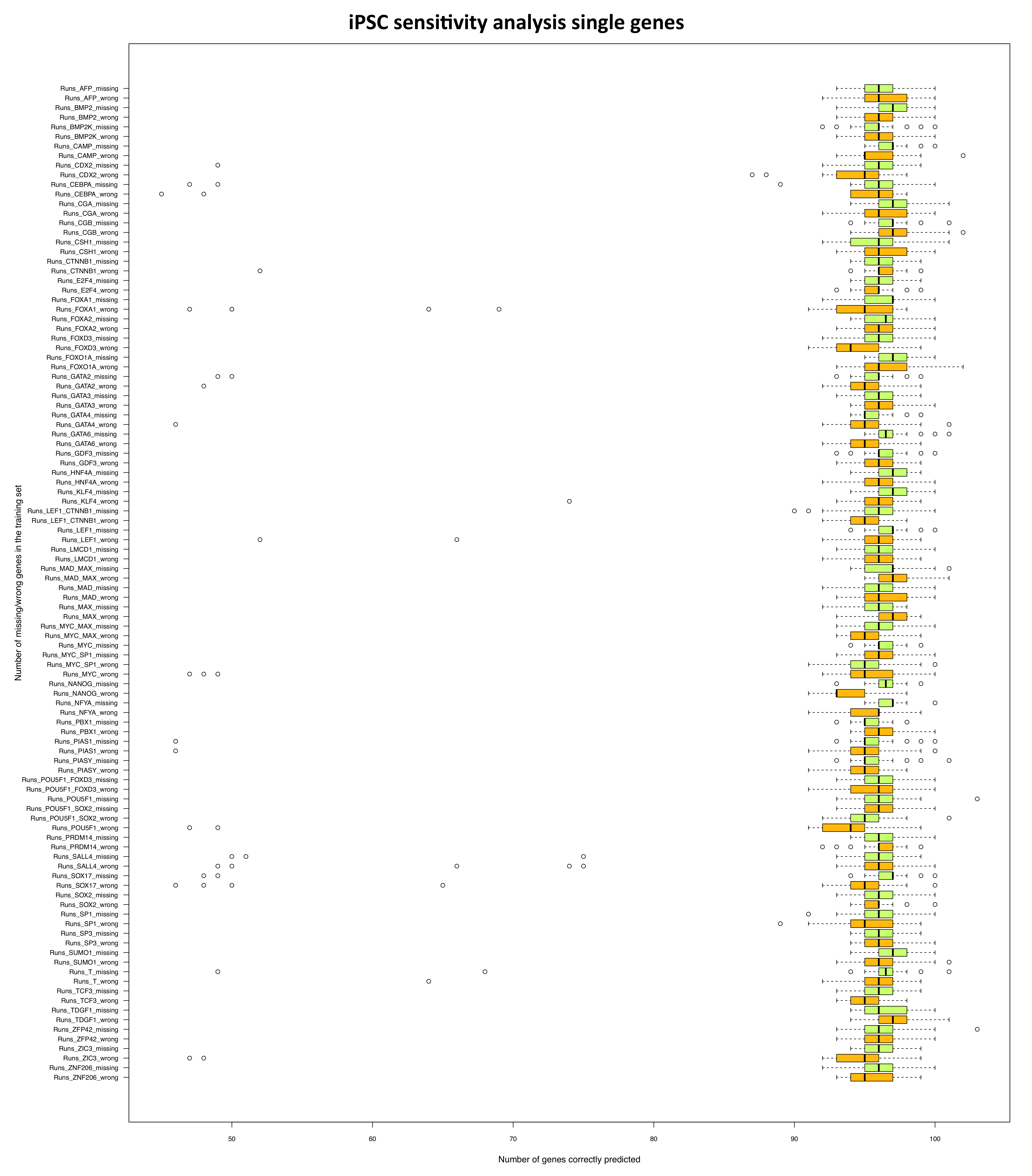 |
| --- |
| **S4 Fig. Sensitivity analysis iPSC single genes.** The boxplot summarize the distribution of scores of contextualized networks when either missing (in green) or wrong (orange) information about specific genes is given to PRUNET. The results indicated that NANOG the most sensitive gene of the model. |

| **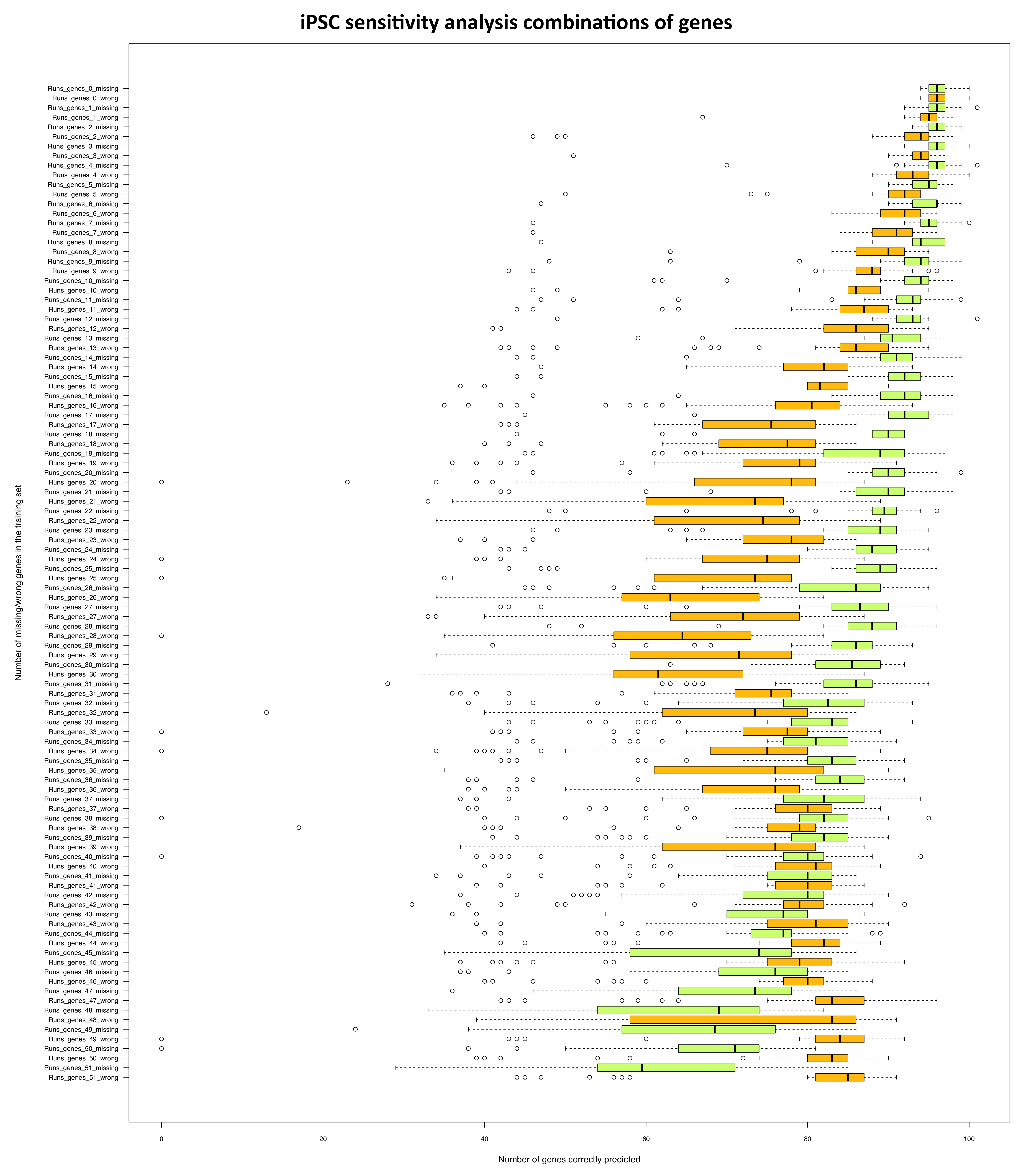** |
| --- |
| **S5 Fig. Sensitivity analysis iPSC combinations of genes.** The boxplot summarize the distribution of scores of contextualized networks when either missing (in green) or wrong (orange) information about combinations randomly selected is given to PRUNET. |

| **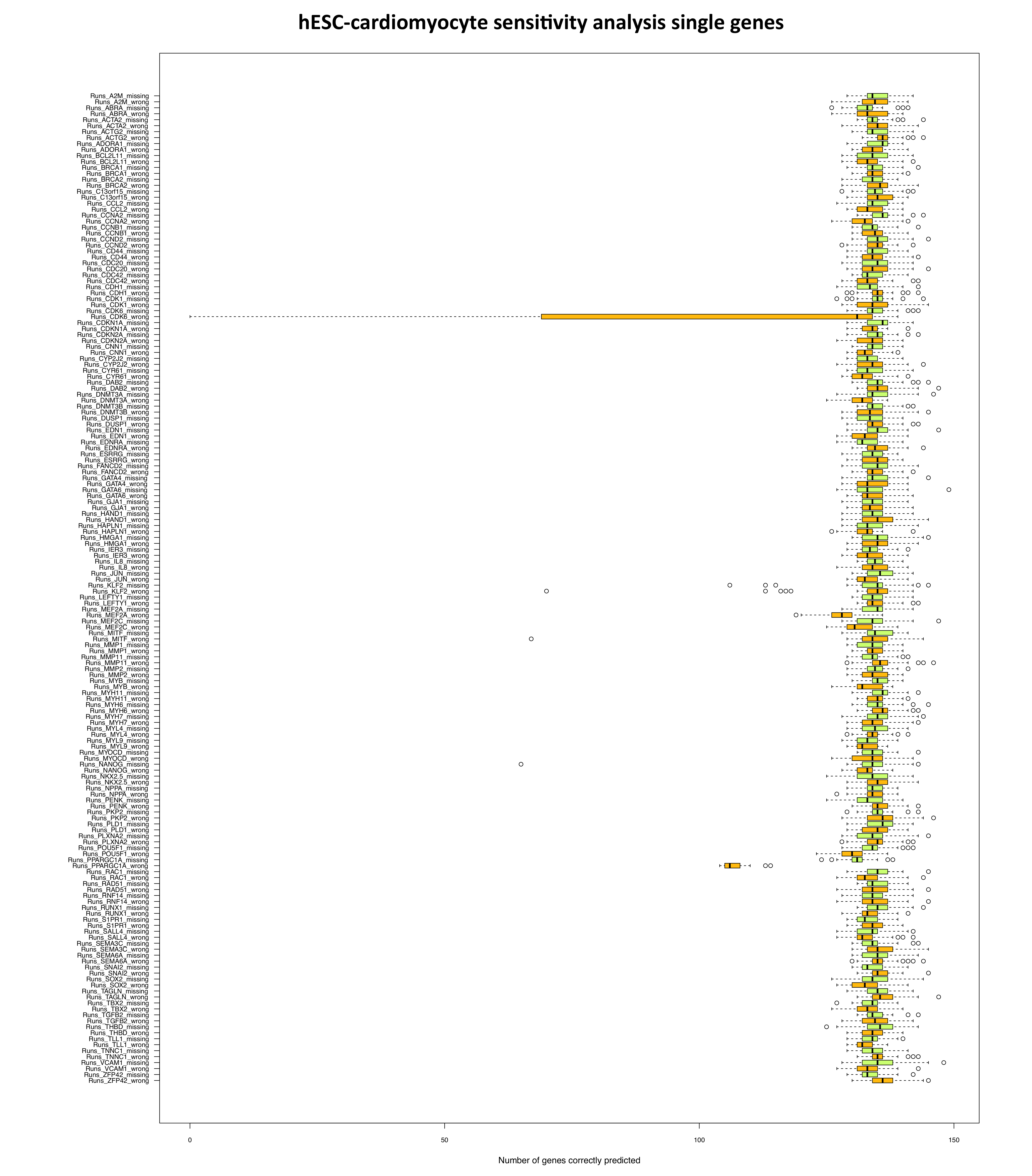** |
| --- |
| **S6 Fig. Sensitivity analysis hESC-cardiomyocyte single genes.** The boxplot summarize the distribution of scores of contextualized networks when either missing (in green) or wrong (orange) information about specific genes is given to PRUNET. The results indicated that POU5F1 was the most sensitive gene of the model. |

| **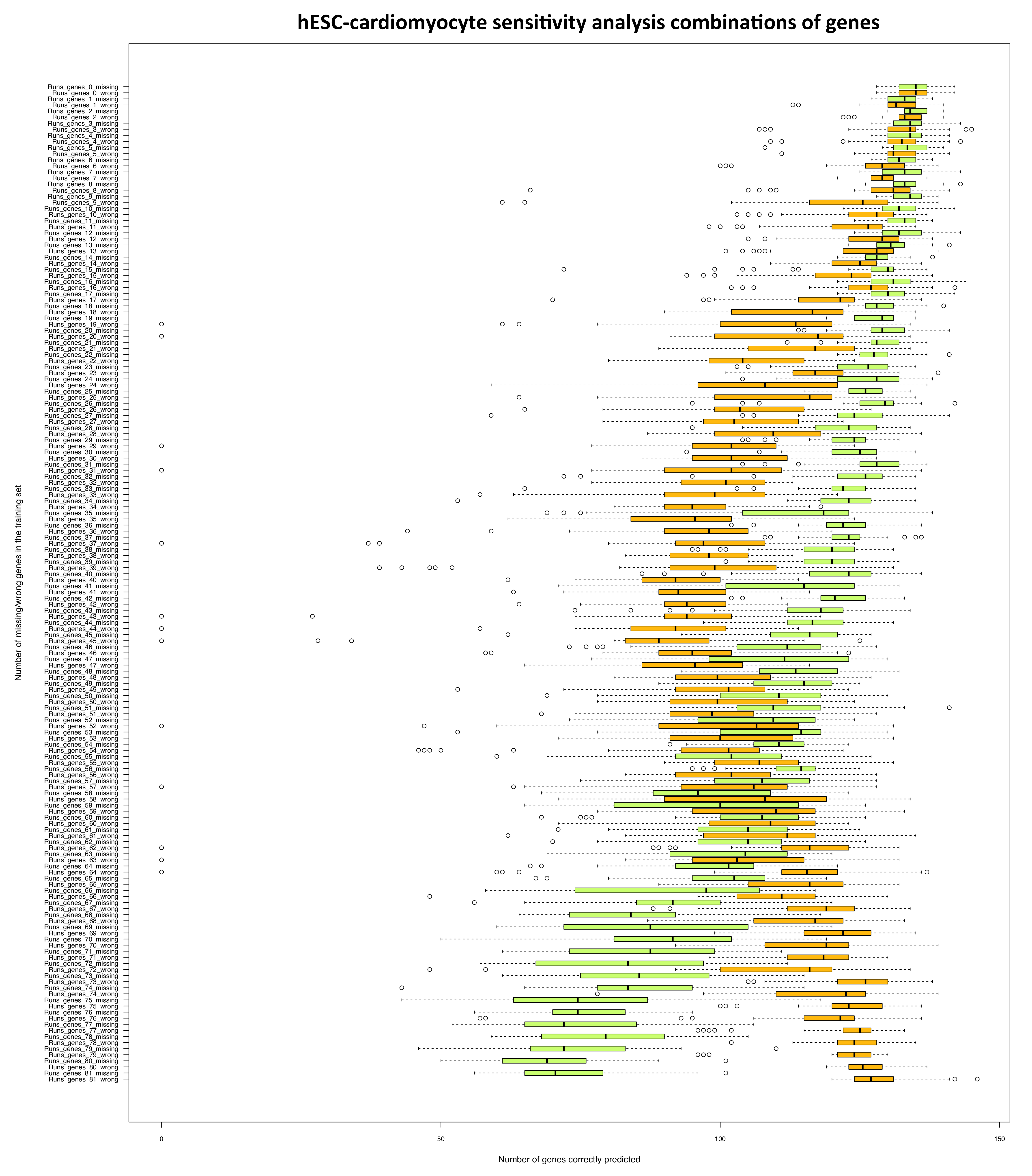** |
| --- |
| **S7 Fig. Sensitivity analysis hESC-cardiomyocyte combinations of genes.** The boxplot summarize the distribution of scores of contextualized networks when either missing (in green) or wrong (orange) information about combinations randomly selected is given to PRUNET. |
